# Effects of Mass Change on Liquid–Liquid Phase Separation of the RNA-Binding Protein Fused in Sarcoma

**DOI:** 10.3390/biom13040625

**Published:** 2023-03-30

**Authors:** Weiqian Dong, Chun Tang, Wen-Ting Chu, Erkang Wang, Jin Wang

**Affiliations:** 1 State Key Laboratory of Electroanalytical Chemistry, Changchun Institute of Applied Chemistry, Chinese Academy of Sciences, Changchun 130022, China; 2 School of Applied Chemistry and Engineering, University of Science and Technology of China, Hefei 230029, China; 3 Beijing National Laboratory for Molecular Sciences, College of Chemistry and Molecular Engineering, Beijing 100871, China; 4 Peking-Tsinghua Center for Life Sciences, Academy for Advanced Interdisciplinary Studies, Peking University, Beijing 100871, China; 5 Department of Chemistry and Physics, Stony Brook University, Stony Brook, NY 11794-3400, USA

**Keywords:** liquid–liquid phase separation, coarse-grained simulation, molecular mass, LLPS stability, FUS

## Abstract

In recent years, many experimental and theoretical studies of protein liquid–liquid phase separation (LLPS) have shown its important role in the processes of physiology and pathology. However, there is a lack of definite information on the regulation mechanism of LLPS in vital activities. Recently, we found that the intrinsically disordered proteins with the insertion/deletion of a non-interacting peptide segment or upon isotope replacement could form droplets, and the LLPS states are different from the proteins without those. We believed that there is an opportunity to decipher the LLPS mechanism with the mass change perspective. To investigate the effect of molecular mass on LLPS, we developed a coarse-grained model with different bead masses, including mass 1.0, mass 1.1, mass 1.2, mass 1.3, and mass 1.5 in atomic units or with the insertion of a non-interacting peptide (10 aa) and performed molecular dynamic simulations. Consequently, we found that the mass increase promotes the LLPS stability, which is based on decreasing the z motion rate and increasing the density and the inter-chain interaction of droplets. This insight into LLPS by mass change paves the way for the regulation and relevant diseases on LLPS.

## 1. Introduction

Liquid–liquid phase separation (LLPS) plays an essential role in cell survival, which is a physicochemical phenomenon in which a solution of proteins and/or nucleic acids concentrates into a distinct, dense phase in equilibrium with a dilute phase depleted in macromolecules [1,2]. Although the concept of membraneless compartments inside cells such as the nucleolus were described as early as the 1830s [3], recently mounting evidence on the wide-ranging roles that biomolecular condensates, including the nucleolus, nuclear speckles, stress granules, Cajal bodies, and P bodies [4,5,6,7], are viewed as critical in regulating diverse cellular function have reignited interest in the behaviors of biological LLPS [8]. The functions of biomolecular condensates referred to as membraneless organelles (MLOs) include cell signaling, nuclear transcription, RNA splicing and processing, and DNA sensing and damage repair [3,5,6,7,9,10,11,12,13]. Importantly, dysregulation of LLPS has been associated with the pathogenesis of neurodegenerative diseases, including amyotrophic lateral sclerosis (ALS), frontotemporal dementia (FTD), and Alzheimer’s, Parkinson’s, and Huntington’s diseases [6,14,15,16,17,18]. Although there is no study which can decipher conclusively the cellular and pathologic basis of the diseases, the unifying observation of abnormal protein inclusions in postmortem tissue may suggest that one well-characterized cellular feature of neurodegenerative disease is the deposition of protein aggregates in affected brain regions [19].

Proteins that undergo LLPS tend to be the intrinsically disordered protein (IDP) or contain the intrinsically disordered region (IDR) which lacks a defined secondary structure [20]. Uversky, Dunker et al. opened the door to the investigation of IDPs [21,22], and Uversky et al. firstly proposed that IDPs serve as important drivers of intracellular LLPS based on the comprehensive assessment of protein intrinsic disorder predisposition by in silico predictors [23]. Recently, Uversky et al. developed a novel web platform named BIAPSS, which can uncover the sequence-encoded signals of proteins capable of undergoing LLPS [24]. IDRs are typically enriched in charged, polar, and/or aromatic amino acids and contain amino acids such as glycine and proline that may convey some structural information [6]. Based on the specific composition and the abundance of amino acids, IDRs can be further classified into arginine/glycine-rich (RG/RGG) domains, phenylalanine/glycine (FG) domains, and prion-like domains (PrLDs), which respectively engage in weak multivalent interactions responsible for driving phase transitions [6].

RNA-binding protein fused in sarcoma (FUS) is a canonical IDP for neurodegenerative diseases, which is mis-localized to cytoplasmic inclusions in degenerating neurons with the onset of ALS and FTD [25]. Furthermore, the FUS is an important model for investigating the LLPS behavior of IDPs/IDRs, and there are abundant studies for LLPS using the FUS model. Some functional MLOs containing FUS are modulated by the recognition of FUS to special RNA fragments [6,16,26]. Kang et al. found that the LLPS of FUS whose aggregation leads to ALS/FTD is enhanced at low concentrations for ATP but is dissolved at high concentrations [27]. In addition, the MD simulations results of Aida et al. have revealed that ATP affects LLPS of FUS by promoting both hydration and solubilization of FUS [28]. On the other hand, Levone et al. found that FUS-dependent LLPS is the requirement of the activation of the cellular DNA damage response (DDR) [11]. The studies of Lao et al. have shown in atomistic detail how phosphorylation inhibits FUS LLPS and reverses the FUS gel/solid phase toward the liquid phase [29]. Bock et al. found that N-terminal acetylation of FUS LC promotes phase separation and reduces aggregation in *E. coli* [30]. Yoshizawa et al. found that the importin karyopherin-beta 2/transportin-1 inhibits LLPS of FUS [31]. In addition, some studies found that environmental factors including pH, molecular crowder [32], temperature [33,34], salt concentration [35], and osmotic pressure [36,37] also affect FUS LLPS and aggregation.

At a given temperature T, higher mass leads to slower thermal motions for the beads, which shows the average effect of mass at the macroscopic level. However, it is unclear how the mass of IDP affects LLPS at the molecular level. Three common techniques to study IDPS that form condensates are solution NMR spectroscopy, small-angle X-ray scattering (SAXS), and Förster resonance energy transfer (FRET), but all of these are relatively low-resolution methods [38,39]. Due to the lack of persistent secondary structures, multiple fuzzy conformations, difficulty in aligning low-complexity regions (LCRs), of obtaining structural properties of droplets, and of choosing appropriate mutations for IDPs, our current molecular understanding of LLPS through experimental approaches is still restrictive [39]. In comparison, molecular dynamics (MD) simulations provide an insightful route to characterize the dynamics of LLPS on atomic and microsecond scales and to generate detailed information on conformational ensembles of IDPs and the contacts formed within a condensate composed of IDP molecules [40]. Best et al. developed a coarse-grained simulation method to determine thermodynamic phase diagrams of IDPs [41] and characterized phase boundaries and material properties for 20 diverse IDP sequences [42]. Additionally, Best et al. used the coarse-grained models to determine the hydrophobicity scale, which can predict LLPS of a given protein and confirms the importance of pi–pi interactions in LLPS [43]. Uversky et al. demonstrated that conformational dynamics of IDPs can rewire the regulatory networks by combining experimental measurements with coarse-grained simulations [44]. There are two ways that the molecular mass of an IDP would change: isotope replacement or the insertion/deletion of a non-interacting peptide segment. In this study, both methods are applied. So, to elucidate the accurate mass effect on LLPS at molecular level, we develop different models based on the two segments of FUS, including a prion-like domain of 50-residue length and an RGG domain of 50-residue length and perform coarse-grained MD simulations. Our results provide the detailed mechanism how IDPs mass change affects the LLPS behaviors of FUS segments.

## 2. Materials and Methods

### 2.1. Simulation System

**Figure 1:**
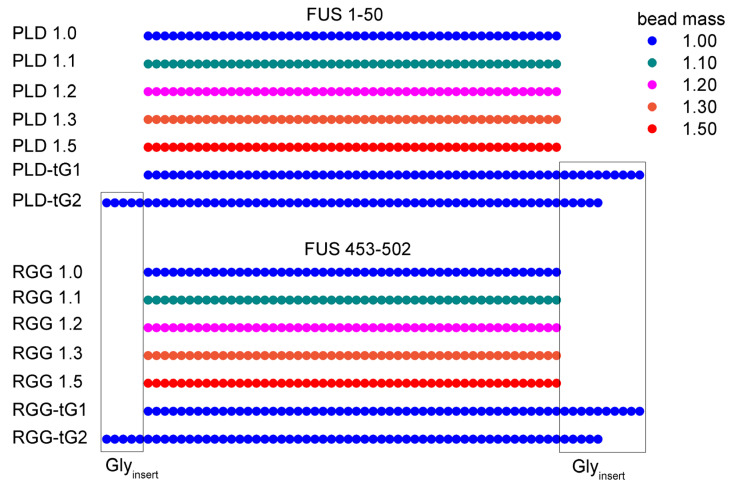
The schematic diagram of different FUS chain models. The different colors represent different bead masses. The beads within rectangle area represent inserted glycine residues.

To our knowledge, there is an effect of chain length on phase diagram [41], and Best et al. found that the results of the slab method and Monte Carlo method of sampling phase coexistence are in good agreement, especially for the proteins whose chain length is equal to 20 or 50 [42]. The major splicing isoform of FUS consists of 526 residues, as reported, and the intrinsically disordered domain of proteins is crucial for the formation of droplets for FUS proteins, which are prion-like domain, RGG1 domain, RGG2 domain, and RGG3 domain [45]. Considering RGG3 domain (FUS 453-501) is about 50 aa, in this study, we selected two amino acid sequences for comparison, which were truncated as 1–50 residue and 453–502 residue in the FUS amino acid sequence, denoted as PLD and RGG, respectively. Next, molecular dynamics simulations with coarse-grained and slab models [41] are used to capture the behavior of the IDPs with or without LLPS. We used the tool of SMOG website to simplify the process of transforming the PDB structure to the coarse-grained model provided for GROMACS [46]. In our coarse-grained model, each amino acid residue is represented by a single bead, using its C_α_ position, and all beads of a protein sequence have the same mass (shown in Figure 1) [47]. To investigate the effort of isotope labels for the behavior of IDPs in LLPS, the mass of each bead of normal protein is set as 1.00. In contrast, the bead mass of isotope-labeled protein is set as 1.20. As shown in Figure 1, normal PLD chain model (PLD 1.0), isotope-labeled PLD chain model (PLD 1.2), normal RGG chain model (RGG 1.0), and isotope-labeled RGG chain model (RGG 1.2) were treated as four simulation systems. In addition, we supplemented mass 1.1, mass 1.3, and mass 1.5 systems to verify conclusions from the comparison of the normal FUS (mass 1.0) systems and the isotope-labeled FUS (mass 1.2) systems. In each system, 200 identical FUS chains (*n* = 200) were added in the simulation box. Hence, the total number of beads in each system is 10,000.

In order to investigate the effect of IDPs with the insertion/deletion of a non-interacting peptide segment to LLPS from mass change perspective, we constructed four models, as shown in Figure 1, referred to as PLD-tG1, PLD-tG2, RGG-tG1, RGG-tG2. PLD-tG1, and RGG-tG1, adding 10 glycine amino acids to the end of the PLD sequence and RGG sequence. PLD-tG2 and RGG-tG2 add 5 glycine amino acids to the head and the end of both the PLD sequence and RGG sequence. In each system, 200 identical FUS chains with glycines insertion (*n* = 200) were added in the simulation box. Hence the total number of beads in each system is 12,000. The masses of these four models are the same as the mass 1.2 system models.

**Figure 2:**
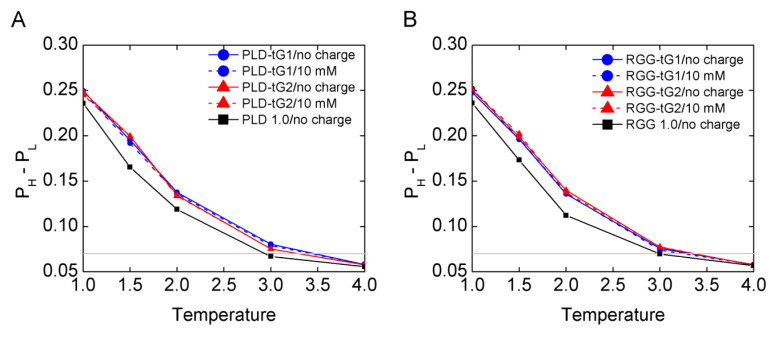
P_H_ − P_L_ of PLD-tG (**A**) and RGG-tG (**B**) in different solvents changes with temperature when *ε*-insert=0.00001 kJ/mol. The P_H_ and P_L_ values are calculated with the last 1000 τ simulation data as P_H_ − P_L_ of all the simulations reaches equilibrium after 3000 τ (see Figures S4–S7). The gray line is P_H_ − P_L_ = 0.07.

In this study, our model incorporates a potential energy function including bonded potential, 12-6 Lennard-Jones (LJ) potential, and Debye-Hückel potential to represent bonding, backbone rigidity, Van der Waals interactions as well as electrostatic interactions, where the bonded potential is classical harmonic model and is given by

(1) $$U_{b}(r_{ij})=K_{b}{\left(r_{ij}-r_{0}\right)}^{2}$$

where the bond constant $K_{b}$ is taken to be 20,000 kJ∙nm^−2^∙mol^−1^ and the equilibrium bond length $r_{0}$ is equal to 3.8 Å. The standard Lennard − Jones potential is given by

(2) $$U_{LJ}=\varepsilon \left[{\left(\frac{\sigma }{r}\right)}^{12}-{\left(\frac{\sigma }{r}\right)}^{6}\right]$$

where the parameter *σ* is the “finite distance”, *σ_ij_* is the optimal distance between beads *i* and *j* that are in contact with each other. We consider the parameter σ equals a constant; that is, the *σ* is 10 Å (about 2.6 a, a = 3.8 Å is the mean bond length). In addition, we performed a series of Langevin dynamics simulations on isolated normal RGG and found that when the parameter *ε* is equal to 0.001, 0.01, 0.1 kJ/mol (*σ* = 1.0 nm), respectively, the head-to-tail distance (D) results are similar and show that the isolated protein chain is in the disorder state (see Supplementary Materials Figure S1). Considering our simulations are not completely quantitative, only qualitative, the energy parameter *ε* is set as 0.001 kJ/mol to make sure to capture the behavior of FUS chains in LLPS and LLPS disappears eventually [48]. For the glycine-inserted FUS chains, there are two *ε* parameters, one is the scale of LJ interaction between the residues of the FUS chains, which is set as 0.001 kJ/mol. The other is the scale of LJ interaction between an inserted residue and another residue (referred as *ε*-insert) and is set as 0.00001 kJ/mol, which indicates that the inserted peptide is non-interacting. We performed a series of test simulations with three *ε*-insert parameters for glycine-inserted FUS chains and found the *ε*-insert parameters as 0.0001, 0.00001 or 0.000001. The results are all similar (shown in Figure 2, Figures S2 and S3). This validated that the effect of the interaction changed by glycine-inserted peptide to LLPS is negligible.

In addition, the Debye − Hückel potential is given by

(3) $$V_{\mathrm{Debye}-\mathrm{Hückel}}=K_{coulomb}B\left(\kappa \right)\underset{i,j}{\sum }\frac{q_{i}q_{j}\exp\left(-\kappa r_{ij}\right)}{\epsilon r_{ij}}$$

where *K_coulomb_* = 138.94 kJ∙mol^−1^∙nm∙e^−2^ is the electric conversion factor; *B*(*κ*) is the salt-dependent coefficient; *κ*^−1^ is the Debye screening length, which is directly dependent on the solvent ionic strength (IS)/salt concentration *C_salt_* ($\kappa \approx 3.2\sqrt{C_{salt}}$); *ϵ* is the dielectric constant, which was set to 80 during the simulations to mimic the solvent medium (water); *q_i_* and *q_j_* are the charges of beads i and j. In our model, aspartic acid and glutamic acid have a negative charge, *q* = −1, and lysine and arginine have a positive charge, *q* = +1. Other residues were set to *q* = 0. Thus, the PLD chain possesses 2 negative charges and the RGG chain possesses 6 negative charges and 9 positive charges. In order to investigate the role of electrostatic interaction for the phase separation with changed beads mass, we consider two extreme conditions with one where salt concentration is 10 mM (*C_salt_* = 0.01 M), which represents there being almost no effect of electrostatic screening, and the other where there is no charge interaction.

After the initial equilibrium (10 ns NVT and 10 ns NPT simulations), we changed the simulation box by elongating the z dimension to 300 nm (z = 300 nm) and for 10,000 bead systems shortening both the x and y to 31 nm (x = 31 nm, y = 31 nm) or for 12,000 bead systems shortening both the x and y to 34 nm (x = 34 nm, y = 34 nm) [41]. Compared with the cubic box approach [49], using slab method reduces the simulation cost and does not affect results [41,50]. Then, 5 μs long-time simulations are conducted to all FUS chains systems at multiple temperatures with two strength-of-charge interactions using constant temperature and volume with a Langevin thermostat with 2.0 fs time step and 1.0 ps^−1^ friction coefficient. In order to cover the overall process from LLPS to phase-separation disappearance, we used a series temperature from 100 K to 400 K (100, 150, 200, 300, and 400 K) in Gromacs. For the reduced unit in the coarse-grained model, we set the unit temperature (T_0_) and unit time (τ) to 100 K and 1 ns in Gromacs. As a result, the simulation temperatures correspond to 1.0, 1.5, 2.0, 3.0, and 4.0 T_0_, and the simulation length of each trajectory corresponds to 5000 τ. In the simulation, all the scales including the length scale, time scale, mass scale, and energy scale are based on theory and used as reduced unit, so the simulation temperature/time cannot be equal to the real temperature directly [51,52]. In order to avoid misunderstanding, we did not mention K in following figures involving the temperature.

### 2.2. Data Analysis

We introduced the maximum difference of local density of beads in the box to describe the extent of phase-separation. The local density of beads is determined by the proportion (P_γ_ = m_γ_/N, γ = 1…30) of the bead number (denote as m_γ_) of each window in the amount (N) of beads of the box, where the window is the order coordinate set by cutting the z axis into 30 windows (γ, the length of γ is 10 nm) and then clustering each bead of the specified window according to the z coordinate of the bead. For the bead density distribution function of z, we calculate the difference between the highest (P_H_) and the lowest (P_L_) values. When LLPS occurs, the protein solution emerges, and demixing and a phenome of the condensed-phase and dilute-phase coexisting in solution is observed, which can be characterized by the difference (P_H_-P_L_) of the densities of the two coexisting phases and the value greater than LLPS more obviously.

We introduced the z motion rate of the chains in each model to investigate the role of variant bead mass in the velocity perspective as the same reason to calculate the flux for the droplet boundary. In order to distinguish condensed-phase vs. dilute-phase simply based on a boundary line, we set the center of the system (condensed-phase with LLPS) at the zero point of x, y, and z axes and took the distance/displacement of each chain (center of mass (CM)) to the zero point on the z axis (|*z*|*_n_* = |*z_n_* − *z*_0_|, *n* = 1…200) as the coordinate of each chain. Hence, the condensed-phase is below and the dilute-phase is above for a boundary line when LLPS occurs. As the same as above, we cut the |z| into 30 windows as reaction coordinate. Each window length is 5 nm. Subsequently, the z motion rate is calculated by averaging the change rates of z coordinate of each chain in the box. The *z* motion rate equation is given by

(4) $$v_{m}=\frac{\sum_{t_{0}}^{t_{m}}\sum_{n=1}^{200}\left(\left|z_{n}\left(t_{m+1}\right)-z_{n}\left(t_{m}\right)\right|\right)}{200\times 2000\Delta t}$$

where $t_{0}$ is 4000 τ, $t_{m}$ limit is 5000 τ, $z_{n}\left(t_{m}\right)$ is the *z* coordinate of the *n* chain at time *t_m_*, $\Delta t=\left(t_{m+1}-t_{m}\right)$ is 0.5 τ.

The flux equation is given by

(5) $$N_{t}=\frac{\sum_{n=1}^{200}f\left(z_{n}\left(t\right)\right)}{0.5\tau }$$

(6) $$f\left(z_{n}\left(t\right)\right)=\{\begin{array}{c} 1\;,\;\;\left(z_{n}\left(t\right)-\left(2750-z_{bundary-line}\right)\right)\times \left(z_{n}\left(t+1\right)-\left(2750-z_{bundary-line}\right)\right)<0\\ or\;\left(z_{n}\left(t\right)-\left(2750+z_{bundary-line}\right)\right)\times \left(z_{n}\left(t+1\right)-\left(2750+z_{bundary-line}\right)\right)<0\\ or\;z_{n}\left(t\right)\times z_{n}\left(t+1\right)<0\\ 0,\;others \end{array}$$

where $z_{n}\left(t\right)$ is the z coordinate of the *n* chain at time *t*, and $z_{bundary-line}$ is 250 angstroms, which is the distance between the center of condensed-phase and the boundary line.

In addition, we analyzed the electrostatic interactions by calculating the amount of intra-chain electrostatic contact (E_intra_) and the amount of inter-chain electrostatic contact (E_inter_). E_intra_ is defined by the number of intra-chain contacts when the pairwise (C_α_-C_α_) distance between residues of having opposite charge within same chain was less than 12 Å. Similarly, E_inter_ is defined by the number of inter-chain contacts when the pairwise (C_α_-C_α_) distance between residues having opposite charges in different chain was less than 15 Å. Considering the charge amount of the chain for all systems and the charge environment in two salt concentration solvents, only chains containing RGG sequence and in 10 mM salt concentration solvent take possession of E_intra_ and E_inter_.

## 3. Results and Discussion

### 3.1. The Mass Effect on LLPS Stability

**Figure 3:**
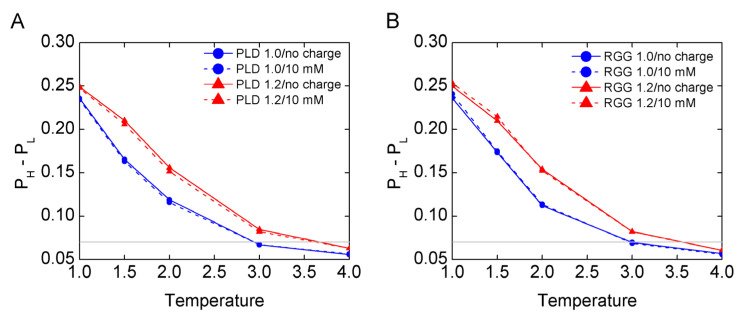
P_H_ − P_L_ of PLD (**A**) and RGG (**B**) with bead mass 1.0 and 1.2 in different solvents changes with temperature. The P_H_ and P_L_ values are calculated with the last 1000 τ simulation data as P_H_ − P_L_ of all the simulations reaches equilibrium after 3000 τ (see Supplementary Materials). The gray line is P_H_ − P_L_ =0.07.

**Figure 4:**
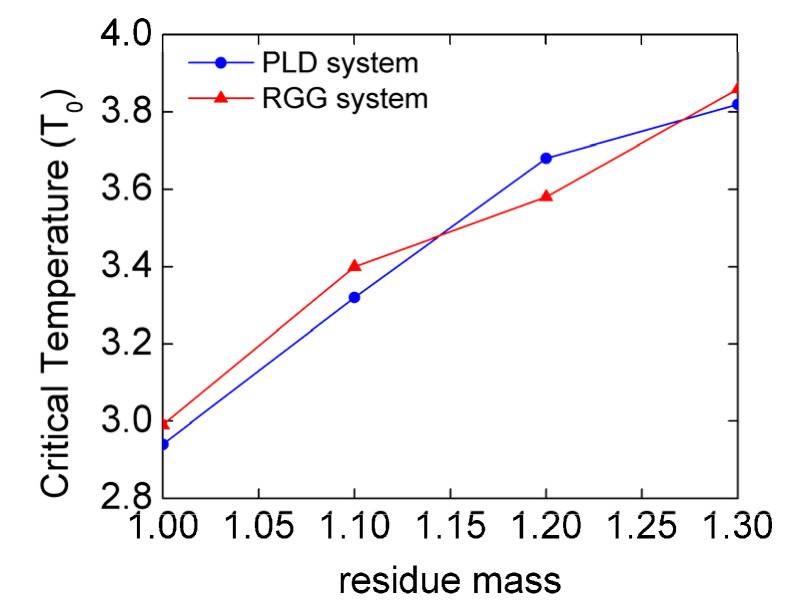
Critical temperature (*T_Cr_*) of LLPS changes with PLD system mass or RGG system mass.

In order to investigate the system phase property, we firstly calculated the difference between P_H_ and P_L_ (P_H_-P_L_) as a function of time τ with an overall 5000 τ simulation time for all the simulations trajectories. As shown in Figures S4–S17, all the simulation models have reached equilibrium after 3000 τ. To further confirm that the systems are in equilibrium, as shown in Figure S18, we calculated the P_H_ − P_L_ average value of every 100 τ simulation time in last 1000 τ data and found that the P_H_ − P_L_ values remain stable over the last 1000 τ simulation time. So, it is safe to say that the simulation systems are in equilibrium in last 1000 τ simulation time. We analyzed the last 1000 τ trajectories, representing the ensemble average values of the equilibrated simulations. As shown in Figure 3 and Figure S19, both normal FUS chains and isotope-labeled FUS chains have a decreasing trend of P_H_ − P_L_ values as the temperature increases. The heavier mass FUS chains systems have a greater P_H_ − P_L_ value at the low temperature (LLPS occurs), which indicates that the LLPS of the heavier mass FUS chains systems are more stable. For example, Figure 3A,B suggest that the temperature of an obvious LLPS (P_H_ − P_L_ > 0.15) for PLD 1.0 chains without charge interaction, PLD 1.0 chains with charge interaction, PLD 1.2 chains without charge interaction, PLD 1.2 chains with charge interaction, RGG 1.0 chains without charge interaction, RGG 1.0 chains with charge interaction, RGG 1.2 chains without charge interaction, and RGG 1.2 chains with charge interaction is below 1.7 T_0_, 1.7 T_0_, 2.0 T_0_, 2.0 T_0_, 1.7 T_0_, 1.7 T_0_, 2.0 T_0_, and 2.0 T_0_, respectively. The FUS chain system’s mass increase from 1.0 to 1.2 enlarges the temperature range about 0.3 T_0_ for the emergence of the obvious LLPS (P_H_ − P_L_ > 0.15), while charge effects on LLPS are not as significant as the mass effect, as shown in Figure 3. At the same time, when the temperature increases, the P_H_ value decreases and the P_L_ value increases (see Figure S20). Furthermore, we found that the LLPS disappears at high temperature (critical temperature, *T_Cr_*) when the density difference is negligible in the protein solution. The critical temperature *T_Cr_* can be obtained by the Flory–Huggins theory or fitting by

(7) $$\rho_{H}-\rho_{L}=A{\left(T_{Cr}-T\right)}^{\beta }$$

where *β* is the critical exponent, and *A* is a protein-specific fitting parameter [41]. When P_H_ − P_L_ = 0, the temperature of the phase diagram equals the critical temperature (*T_Cr_*). However, the absolute zero point of P_H_ − P_L_ cannot be obtained from the simulations. Thus, we set the threshold to be 0.07. LLPS disappears when P_H_ − P_L_ < 0.07. In detail, the *T_Cr_* value is only relevant to residue mass, regardless of the salt concentration in the models. The *T_Cr_* values of PLD 1.0 chains and RGG 1.0 chains are 2.9 T_0_, and those of PLD 1.2 chains and RGG 1.2 chains are about 3.6 T_0_. Hence, it is easy to confirm that the FUS chain system’s mass increase from 1.0 to 1.2 increases the *T_Cr_* by 0.7 T_0_. In addition, as shown in Figure 4, the critical temperature increases as the mass of the systems increases.

As shown in Figure 2, there is no obvious difference of P_H_ − P_L_ value changes with temperature between the FUS segments with different modes of glycine peptide insertion, while the same is true for that in different solvents. Compared with normal FUS chains, the glycine-inserted FUS chains have a greater P_H_ − P_L_ value at the low temperatures (LLPS occurs). This indicates that the LLPS of the glycine-inserted FUS chains are more stable. For example, Figure 2A,B suggest that the temperature of an obvious LLPS (P_H_ − P_L_ > 0.15) for PLD 1.0, PLD-tG1, RGG 1.0, and RGG-tG1 is below 1.7 T_0_, 1.9 T_0_, 1.7 T_0_, and 1.9 T_0_, respectively. The FUS chain with glycine insertion enlarges the temperature range about 0.2 T_0_ for the emergence of the obvious LLPS (P_H_ − P_L_ > 0.15). Additionally, The T_cr_ values of PLD 1.0 chains and RGG 1.0 chains are 2.9 T_0_, and those of PLD-tG1 and RGG-tG1 are about 3.5 T_0_ and 3.3 T_0_, respectively. In summary, both the isotope labeling and the peptide insertion lead to the mass increase and promote the LLPS stability. Thus, the isotope labeling promotes the greater LLPS stability.

### 3.2. The Mechanism of Mass Effect on LLPS

**Figure 5:**
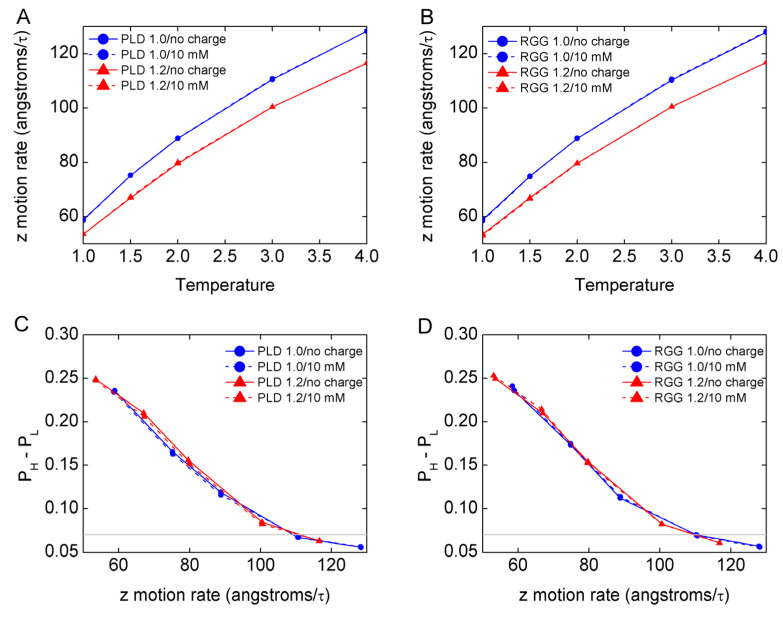
The z motion rate of PLD (**A**) and RGG (**B**) with bead mass 1.0 and 1.2 in different solvents changes with temperature; P_H_ − P_L_ of PLD (**C**) and RGG (**D**) with bead mass 1.0 and 1.2 in different solvents change with z motion rate. The z motion rates are calculated by averaging 200 chains in the box with the last 1000 τ simulation data. The gray line is P_H_ − P_L_ = 0.07.

Considering that each chain undergoes the stochastic dynamics in the simulation, motion and diffusion of beads may slow down when the mass increases from 1.00 to 1.50. In this simulation, to quantify the motion on the z axis of the slab model, we calculated the average z motion rate of all the 200 chains. As shown in Figure 5 and Figure S21, the results suggest that the z motion rate increases as the temperature increases. In addition, the z motion rate of the mass of heavier FUS chains is always smaller than that of the normal FUS chains at the same temperature. For example, at 2.0 T_0_, the z motion rate of PLD 1.2 chains without charge is 79.6 Å/τ, lower than that of PLD 1.0 chains (88.8 Å/τ). As shown in Figure 5C,D, the P_H_ − P_L_ values decrease as the z motion rates increase for normal and isotope-labeled models. The results suggest that the z motion rate is strongly correlated with the stability extent of the LLPS, and lower rate of z motion favors the formation and stability of LLPS.

**Figure 6:**
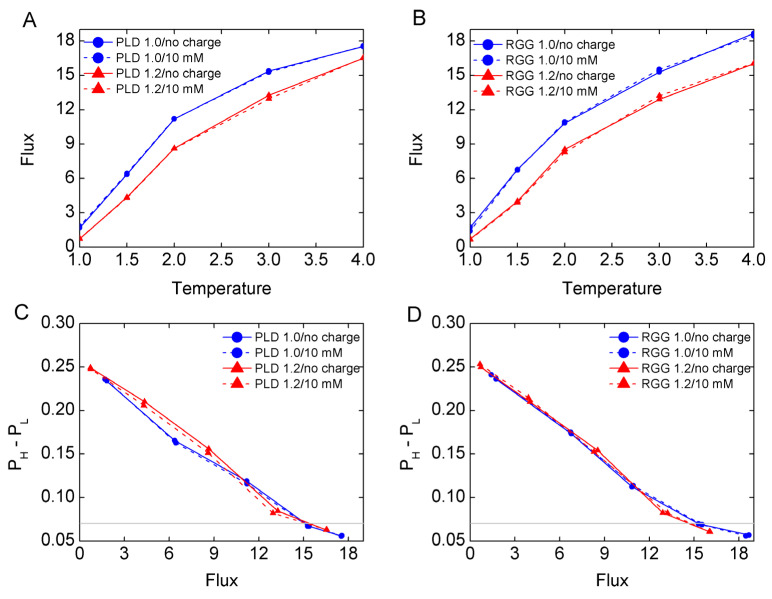
The flux on the boundary line. (**A**) The flux of PLD 1.0 and PLD 1.2 in different solvents changes with temperature. (**B**) The flux of RGG 1.0 and RGG 1.2 in different solvents changes with temperature. (**C**) P_H_ − P_L_ of PLD 1.0 and PLD 1.2 in different solvents change with flux. (**D**) P_H_ − P_L_ of RGG 1.0 and RGG 1.2 in different solvents change with flux. The fluxes are calculated by averaging the last 1000 τ simulation data, and the boundary line is |z| = 25 nm. The gray line is P_H_ − P_L_ = 0.07.

In order to quantify the diffusion of molecules between condensed-phase and dilute-phase, we calculated the average flux of the chains across the boundary line of the condensed-phase during last 1000 τ time. As shown in Figure 6A,B, the flux increases as temperature rises for all models. The flux values of isotope-labeled FUS chains are smaller than that of normal FUS chains at the same temperature. For example, at 2.0 T_0_, the flux of PLD 1.2 chains without charge is 8.6 chains/τ, lower than that of PLD 1.0 chains (11.2 chains/τ). As shown in Figure 6C,D, the P_H_ − P_L_ values decrease as the flux values increase for normal and isotope-labeled models. As shown in Figure S22, we calculated the average flux during the last 100 τ simulation time to confirm that our systems are in equilibrium and the results are reliable. As a result, the flux is strongly correlated with the stability extent of LLPS and lower flux values favor LLPS formation and stability, whose results are equivalent to the z motion rate’s.

As shown in Figure S23A,B, the results suggest that the z motion rate increases as the temperature increases, and there is no obvious difference for different inserted modes and different solvents. In addition, the z motion rate of glycine-inserted FUS chains is always smaller than that of normal FUS chains at the same temperature. For example, at 2.0 T_0_, the z motion rate of PLD-tG1 chains is 80.5 Å/τ, lower than that of PLD 1.0 chains (88.8 Å/τ). As shown in Figure S23C,D, the P_H_ − P_L_ values decrease as the z motion rates increase for glycine-inserted models, which are the same as the isotope-labeled FUS chains.

As shown in Figure S24A,B, the flux increases as the temperature rises for the glycine-inserted models. The flux values of glycine-inserted FUS chains are smaller than that of the normal FUS chains at the same temperature. For example, at 2.0 T_0_, the flux of PLD-tG1 chains is 9.5 chains/τ, lower than that of PLD 1.0 chains (11.2 chains/τ). As shown in Figure S24C,D, the P_H_ − P_L_ values decrease as the flux values increase for the glycine-inserted models.

**Figure 7:**
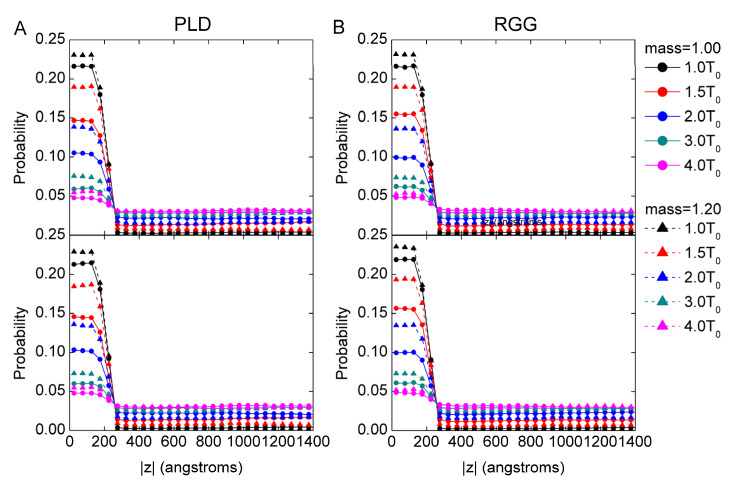
The distribution of probability of PLD (**A**) and RGG (**B**) with bead mass 1.0 and 1.2 as a function of displacement |z|. Data without charges and at 10 mM salt concentration solvent are shown in the upper panels and the bottom panels, respectively. The probability is the average value of window along |z| during the last 1000 τ simulation data.

In addition, we use the distribution of probability of FUS chains as a function of displacement |z| to describe the degree of chain aggregation. As shown in Figure 7 and Figure S25, in the droplet, the probability of the mass heavier of FUS chains is greater than that of normal FUS chains, which indicates that the heavier FUS chains have more concentrated distribution than normal FUS chains at the low temperature (LLPS occurs). For example, at 1.0 T_0_, when |z| = 0 nm, the probability of PLD 1.2 chains is 0.23 greater than that of PLD 1.0 chains (0.21). As shown in Figure S26, the distribution of the probability of glycine-inserted FUS chains is similar to that of the isotope-labeled FUS chains and compared with normal FUS chains. We found that at the lower temperature (LLPS occurs), the probability of glycine-inserted FUS chains distributed in droplets (|z| < 25 nm) is greater than that of normal FUS chains. For example, at 1.0 T_0_, when |z| = 0 nm, the probability of PLD-tG1 chains is 0.23 greater than that of PLD 1.0 chains (0.21). In summary, the mass increase leads to FUS chains being more concentrated in the droplets.

### 3.3. The Effect of Mass Increase on the Conformation of Chains and the Electrostatic Contact

**Figure 8:**
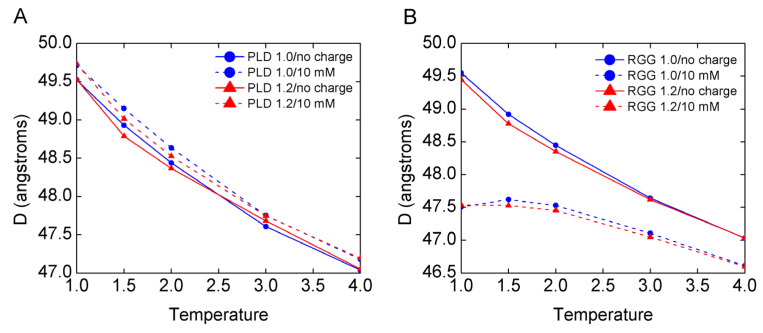
Head-to-tail distance (D) of PLD (**A**) and RGG (**B**) chains with bead mass 1.0 and 1.2 as a function of temperature. The D value is calculated by the mean value of the 200 chains in the system during the last 1000 τ simulation data.

We calculated the mean end-to-end distance (D) of 200 chains to show the conformation changes at different conditions. As shown in Figure 8 and Figure S27, there is a trend that the mean D decreases as the temperature increases in normal and isotope-labeled models. The effect of the mass increases on the mean D can be negligible. This indicates that the mass of chains does not have a significant effect on the conformation of an individual molecule. Intriguingly, the mean D shows difference with different charge patterns. The PLD chains with 10 mM salt concentration solvent have a slightly greater mean D than that without charge interactions. In contrast, the RGG chains with 10 mM salt concentration solvent have a smaller mean D than that without charge interaction. The results suggest that the electrostatic interactions help RGG chains to fold a bit.

**Figure 9:**
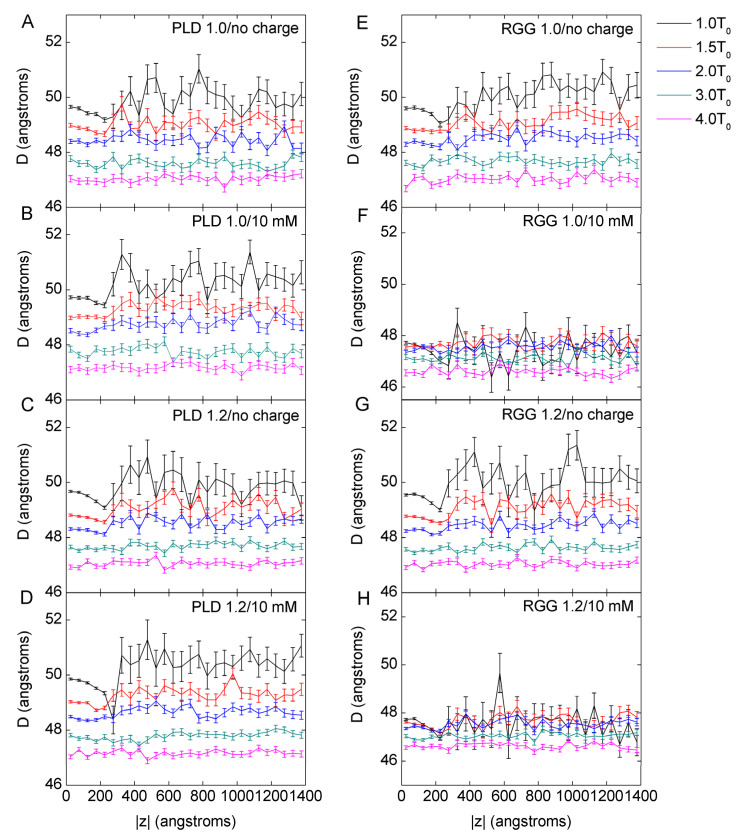
Head-to-tail distance (D) of PLD and RGG as a function of displacement |z|. (**A**) The head-to-tail distance of PLD 1.0 with no charge interaction. (**B**) The head-to-tail distance of PLD 1.0 with 10 mM salt solvent. (**C**) The head-to-tail distance of PLD 1.2 with no charge interaction. (**D**) The head-to-tail distance of PLD 1.2 with 10 mM salt solvent. (**E**) The head-to-tail distance of RGG 1.0 with no charge interaction. (**F**) The head-to-tail distance of RGG 1.0 with 10 mM salt solvent. (**G**) The head-to-tail distance of RGG 1.2 with no charge interaction. (**H**) The head-to-tail distance of RGG 1.2 with 10 mM salt solvent. Mean D of the chains in each window along |z| and standard error (σ) values during the last 1000 τ simulation data are illustrated in this figure. Considering the effect of boundary, the data with displacement z higher than 1400 are not calculated.

In order to distinguish the chain conformations in the condensed-phase and the dilute-phase, we calculated the distributions of D along the displacement |z|. As shown in Figure 9, there is no significant difference between normal and isotope-labeled FUS chains.

For the glycine-inserted FUS chains, we calculated the head-to-tail distance of PLD sequence or RGG sequence excluding the glycine-inserted peptide. As shown in Figure S28, the mean D decreases as the temperature increases in glycine-inserted models. In addition, PLD-tG2 and RGG-tG2 have greater mean D values than that of PLD-tG1 and RGG-tG1, respectively. This indicates that different modes of insertion influence the head-to-tail distance of the FUS chains. Comparing with normal FUS chains and isotope-labeled FUS chains, the mean D values of glycine-inserted FUS chains are greater than that of normal and isotope-labeled FUS chains at the same temperature. We believe that the difference is not caused by the mass increase. In summary, the mass increase hardly affects the mean D of FUS chains and the conformation of FUS chains.

**Figure 10:**
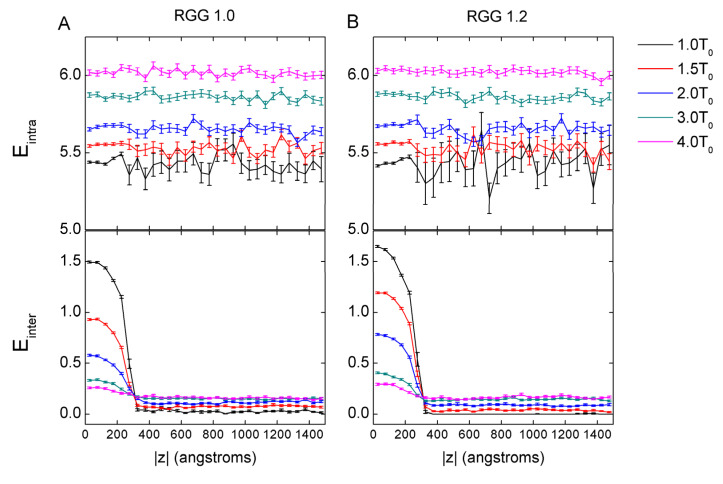
Mean number intra-chain (E_intra_) of and inter-chain (E_inter_) electrostatic contacts of RGG 1.0 (**A**) and RGG 1.2 (**B**) as a function of |z|. Average and standard error values during the last 1000 τ simulation data are illustrated in this figure.

As shown in Figure 10 and Figure S29, there is no significant difference for the intra-chain electrostatic contacts (E_intra_) between the normal RGG chains and the isotope-labeled RGG chains. If the conformation of the chain is curved, the E_intra_ value will be high. Therefore, the E_intra_ value correlates to the D values negatively. In the condensed phase, the E_inter_ value correlates to the local probability of chains positively (as shown in Figure 7, Figure 10, Figures S25 and S29 and P_H_ in Figure S20). The mass increase leads to the increase of the local probability of chains in the condensed-phase (as shown in Figure 7 and Figure S25 and P_H_ in Figure S20). As a result, in the condensed-phase, the E_inter_ values of the isotope-labeled RGG chains are higher than that of the normal RGG chains. For example, E_inter_ of RGG 1.2 chains at |z| = 0 is 1.62 (T = 1.0 T_0_), and by contrast, that of RGG 1.0 chains is 1.49.

As shown in Figure S30, there is no significant difference for the intra-chain electrostatic contacts (E_intra_) and inter-chain (E_inter_) electrostatic contacts between RGG-tG1 chains and RGG-tG2 chains. However, compared with the normal RGG chains and the isotope-labeled RGG chains, both the intra-chain (E_intra_) and inter-chain (E_inter_) electrostatic contacts of RGG-tG1 and RGG-tG2 are smaller. For example, at 1.0 T_0_, when |z| = 0 nm, E_intra_ of RGG-tG1 is 4.32 smaller than that of RGG 1.2 chains (5.42), E_inter_ of RGG-tG1 is 1.21 smaller than that of RGG 1.2 chains (1.62). We believe that the difference is not caused by the mass increase. Considering that the critical temperature of LLPS for RGG-tG chains is greater than that of RGG 1.0 chains and smaller than that of RGG 1.2 chains, we speculated that the electrostatic contact increase is not the main cause of the increased LLPS stability.

## 4. Conclusions

In this study we performed Langevin dynamics simulations to gain insight into the effects and the mechanism of FUS chain mass increase in LLPS. The study was inspired by one of our observations during NMR sample preparation, in which ^15^N, ^13^C, ^2^H-isotope-labeled FUS RGG and another aggregation-prone protein exhibited greater tendency to coacervate than unlabeled protein of the same concentration under the same buffer conditions and temperature. Our simulation results suggest that the mass increase of FUS chain promotes the level of LLPS stability, but different mass increase methods have different devotion to LLPS stability. For the critical transition temperature (*T_Cr_*) where the LLPS start to emerge, the value of RGG 1.2 chains is 0.7 T_0_ higher than that of RGG 1.0 chains, while the value of RGG-tG1 is 0.4 T_0_ higher than that of RGG 1.0 chains. Based on our simulations, the details of how the FUS chain mass change affects the behavior of LLPS at various temperatures and ionic strength are vividly revealed at the molecular level. We found that, in the same environment, the z motion rate of chains of the mass 1.2 system and glycine-inserted system is lower than that of the mass 1.0 system, and the flux of the mass 1.2 system and glycine-inserted system is lower than that of the mass 1.0 system. Therefore, lower z motion rate and lower flux are beneficial to LLPS stability. Furthermore, using the distribution of probability of FUS chains as a function of displacement |z|, the results reveal that the mass increase will increase the degree of chain aggregation at the same temperature, and the chains of the mass 1.2 system and glycine-inserted system both are more concentrated than that of the mass 1.0 system. The mass increase hardly affects the head-to-tail distance (D) of FUS chains. In addition, we have noted that the mass increase by the isotope replacement is favorable to strengthen the inter-chain electrostatic contacts in the condensed-phase, hardly affect the intra-chain electrostatic contacts and the head-to-tail distance of the chains. The effect of the mass increase by glycine insertion on the intra-chain electrostatic contact and the inter-chain electrostatic contact is fuzzy and will be studied in the future. We believe that FUS chain mass increase leads to the increase of the inter-chain electrostatic contact from more concentrated distribution of FUS chains, while the electrostatic contact increase is not the main cause for increased LLPS stability. Consequently, we believe that the mass increase promotes the LLPS stability, which is based on decreasing the z motion rate, increasing the density and the inter-chain interaction of droplets.

Our findings highlight the importance of residue mass change of IDPs on LLPS. Such residue mass change often emerges in the NMR experiments used to explore the information on structures of IDPs. In our study, these changed mass FUS models may provide enlightenment towards understanding the roles of isotope-labeling effects in modulating LLPS. In addition, it is helpful to test more systems with simulation and to elaborate results from the IDP chain mass perspective for investigating the mechanism of LLPS. This may pave the way for ameliorating phase-separation-related pathologies, which will be our future work direction.

## Data Availability

Not applicable.

## Supplementary Materials

The following supporting information can be downloaded at: https://www.mdpi.com/article/10.3390/biom13040625/s1, Figure S1: Head-to-tail distance (D) of RGG with different parameters *ε* (σ = 1.0 nm, *ε* = 0.001, 0.01, 0.1, 0.8 kJ/mol); Figure S2: P_H_ − P_L_ of PLD-tG (A) and RGG-tG (B) in different solvents changes with temperature when *ε*-insert = 0.0001 kJ/mol. The P_H_ and P_L_ values are calculated with the last 1000 τ simulation data as P_H_ − P_L_ of all the simulations reaches equilibrium after 3000 τ. The gray line is P_H_ − P_L_ = 0.07; Figure S3: P_H_ − P_L_ of PLD-tG (A) and RGG-tG (B) in different solvents changes with temperature when *ε*-insert = 0.000001 kJ/mol. The P_H_ and P_L_ values are calculated with the last 1000 τ simulation data as P_H_ − P_L_ of all the simulations reaches equilibrium after 3000 τ. The gray line is P_H_ − P_L_ = 0.07; Figure S4: P_H_ − P_L_ of PLD-tG1 as a function of simulation time. The upper panel shows the simulations without charge interactions (no charge); the bottom panel shows the simulations at 10 mM salt concentration; Figure S5: P_H_ − P_L_ of PLD-tG2 as a function of simulation time. The upper panel shows the simulations without charge interactions (no charge); the bottom panel shows the simulations at 10 mM salt concentration; Figure S6: P_H_ − P_L_ of RGG-tG1 as a function of simulation time. The upper panel shows the simulations without charge interactions (no charge); the bottom panel shows the simulations at 10 mM salt concentration; Figure S7: P_H_ − P_L_ of RGG-tG2 as a function of simulation time. The upper panel shows the simulations without charge interactions (no charge); the bottom panel shows the simulations at 10 mM salt concentration; Figure S8: P_H_ − P_L_ of PLD 1.0 as a function of simulation time. The upper panel shows the simulations without charge interactions (no charge); the bottom panel shows the simulations at 10mM salt concentration; Figure S9: P_H_ − P_L_ of PLD 1.1 as a function of simulation time. The upper panel shows the simulations without charge interactions (no charge); the bottom panel shows the simulations at 10 mM salt concentration; Figure S10: P_H_ − P_L_ of PLD 1.2 as a function of simulation time. The upper panel shows the simulations without charge interactions (no charge); the bottom panel shows the simulations at 10mM salt concentration; Figure S11: P_H_ − P_L_ of PLD 1.3 as a function of simulation time. The upper panel shows the simulations without charge interactions (no charge); the bottom panel shows the simulations at 10 mM salt concentration; Figure S12: P_H_ − P_L_ of PLD 1.5 as a function of simulation time. The upper panel shows the simulations without charge interactions (no charge); the bottom panel shows the simulations at 10 mM salt concentration; Figure S13: P_H_ − P_L_ of RGG 1.0 as a function of simulation time. The upper panel shows the simulations without charge interactions (no charge); the bottom panel shows the simulations at 10mM salt concentration; Figure S14: P_H_ − P_L_ of RGG 1.1 as a function of simulation time. The upper panel shows the simulations without charge interactions (no charge); the bottom panel shows the simulations at 10 mM salt concentration; Figure S15: P_H_ − P_L_ of RGG 1.2 as a function of simulation time. The upper panel shows the simulations without charge interactions (no charge); the bottom panel shows the simulations at 10mM salt concentration; Figure S16: P_H_ − P_L_ of RGG 1.3 as a function of simulation time. The upper panel shows the simulations without charge interactions (no charge); the bottom panel shows the simulations at 10 mM salt concentration; Figure S17: P_H_ − P_L_ of RGG 1.5 as a function of simulation time. The upper panel shows the simulations without charge interactions (no charge); the bottom panel shows the simulations at 10 mM salt concentration; Figure S18: P_H_ − P_L_ average as a function of simulation time. Every point represents the P_H_ − P_L_ average value of 100 τ simulation time; Figure S19: P_H_ − P_L_ of PLD (A) and RGG (B) with bead mass 1.1, 1.3, and 1.5 in different solvents changes with temperature. The P_H_ and P_L_ values are calculated with the last 1000 τ simulation data as P_H_ − P_L_ of all the simulations reaches equilibrium after 3000 τ (see Supplementary Materials). The gray line is P_H_ − P_L_ = 0.07; Figure S20: Phase diagrams of PLD (A) and RGG (B) with P_H_ and P_L_ as a function of temperature. (A) Phase diagrams of PLD 1.0 and PLD 1.2 at different solvents. (B) Phase diagrams of RGG 1.0 and RGG 1.2 at different solvents. Here, P_H_ and P_L_ are the highest and the lowest points of FUS segment residue distribution along the |z| axis; Figure S21: The z motion rate of PLD (A) and RGG (B) with bead mass 1.1, 1.3, and 1.5 in different solvents changes with temperature. The z motion rates are calculated by averaging 200 chains in the box with the last 1000 τ simulation data; Figure S22: The flux of last 100 τ on the boundary line. (A) The flux of PLD 1.0 and PLD 1.2 in different solvents changes with temperature. (B) The flux of RGG 1.0 and RGG 1.2 in different solvents changes with temperature. (C) P_H_ − P_L_ of PLD 1.0 and PLD 1.2 in different solvents changes with flux. (D) P_H_ − P_L_ of RGG 1.0 and RGG 1.2 in different solvents changes with flux. The fluxes are calculated by averaging the last 100 τ simulation data, and the boundary line is |z| = 25 nm. The gray line is P_H_ − P_L_ = 0.07; Figure S23: The z motion rate of PLD-tG (A) and RGG-tG (B) in different solvents changes with temperature; P_H_ − P_L_ of PLD-tG (C) and RGG-tG (D) in different solvents changes with z motion rate. The z motion rates are calculated by averaging 200 chains in the box with the last 1000 τ simulation data. The gray line is P_H_ − P_L_ = 0.07; Figure S24: The flux on the boundary line. (A) The flux of PLD-tG in different solvents changes with temperature. (B) The flux of RGG-tG in different solvents changes with temperature. (C) P_H_ − P_L_ of PLD-tG in different solvents changes with flux. (D) P_H_ − P_L_ of RGG-tG in different solvents changes with flux. The fluxes are calculated by averaging the last 1000 τ simulation data, and the boundary line is |z| = 25 nm. The gray line is P_H_ − P_L_ = 0.07; Figure S25: The distribution of probability of PLD (A) and RGG (B) with bead mass 1.1 and 1.3 as a function of displacement |z|. Data without charges and at 10mM salt concentration solvent are shown in the up panels and the bottom panels, respectively. The probability is the average value of window along |z| during the last 1000 τ simulation data; Figure S26: The distribution of probability of PLD-tG (A) and RGG-tG (B) as a function of displacement |z|. Data without charges and at 10 mM salt concentration solvent are shown in the up panels and the bottom panels, respectively. The probability is the average value of window along |z| during the last 1000 τ simulation data; Figure S27: Head-to-tail distance (D) of PLD (A) and RGG (B) chains with bead mass 1.1, 1.3 and 1.5 as a function of temperature. The D value is calculated by the mean value of the 200 chains in the system during the last 1000 τ simulation data; Figure S28: Head-to-tail distance (D) of PLD-tG (A) and RGG-tG (B) chains as a function of temperature. The D value is calculated by the mean value of the 200 chains in the system during the last 1000 τ simulation data; Figure S29: Mean number intra-chain (E_intra_) of and inter-chain (E_inter_) electrostatic contacts of RGG 1.1 (A) and RGG 1.3 (B) as a function of |z|. Average and standard error values during the last 1000 τ simulation data are illustrated in this figure; Figure S30: Mean number intra-chain (E_intra_) and inter-chain (E_inter_) electrostatic contacts of RGG-tG1 (A) and RGG-tG2 (B) as a function of |z|. Average and standard error values during the last 1000 τ simulation data are illustrated in this figure.
