# Supplementary figures and images for: Effects of Mass Change on Liquid–Liquid Phase Separation of the RNA-Binding Protein Fused in Sarcoma
Source: Biomolecules. 2023 Mar 30;13(4):625. doi: 10.3390/biom13040625 (PMC10135815; doi:10.3390/biom13040625)

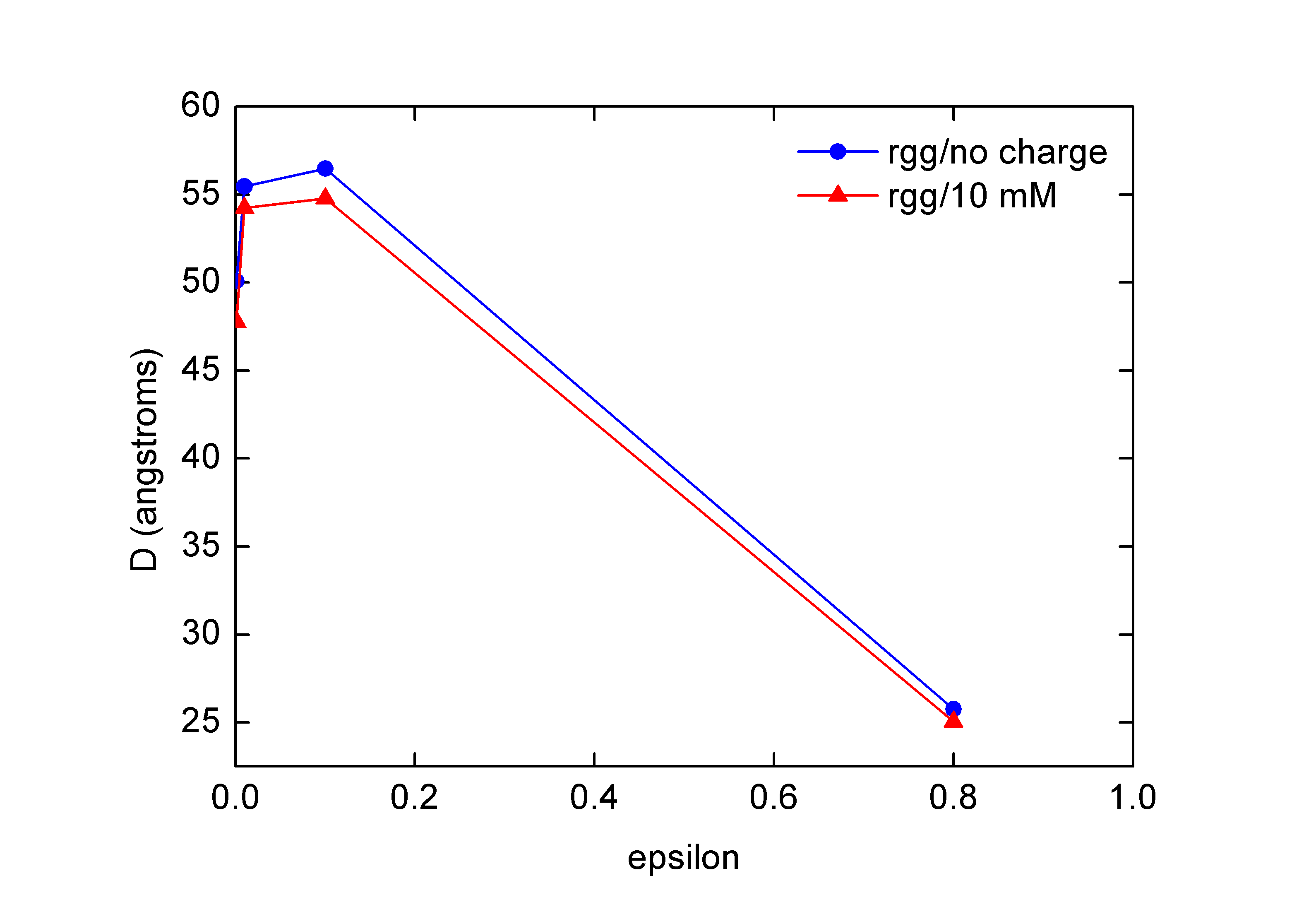

Supplement: Supplementary file 1 [file biomolecules-13-00625-s001.zip › FigS1.Parameter epsilon.png]

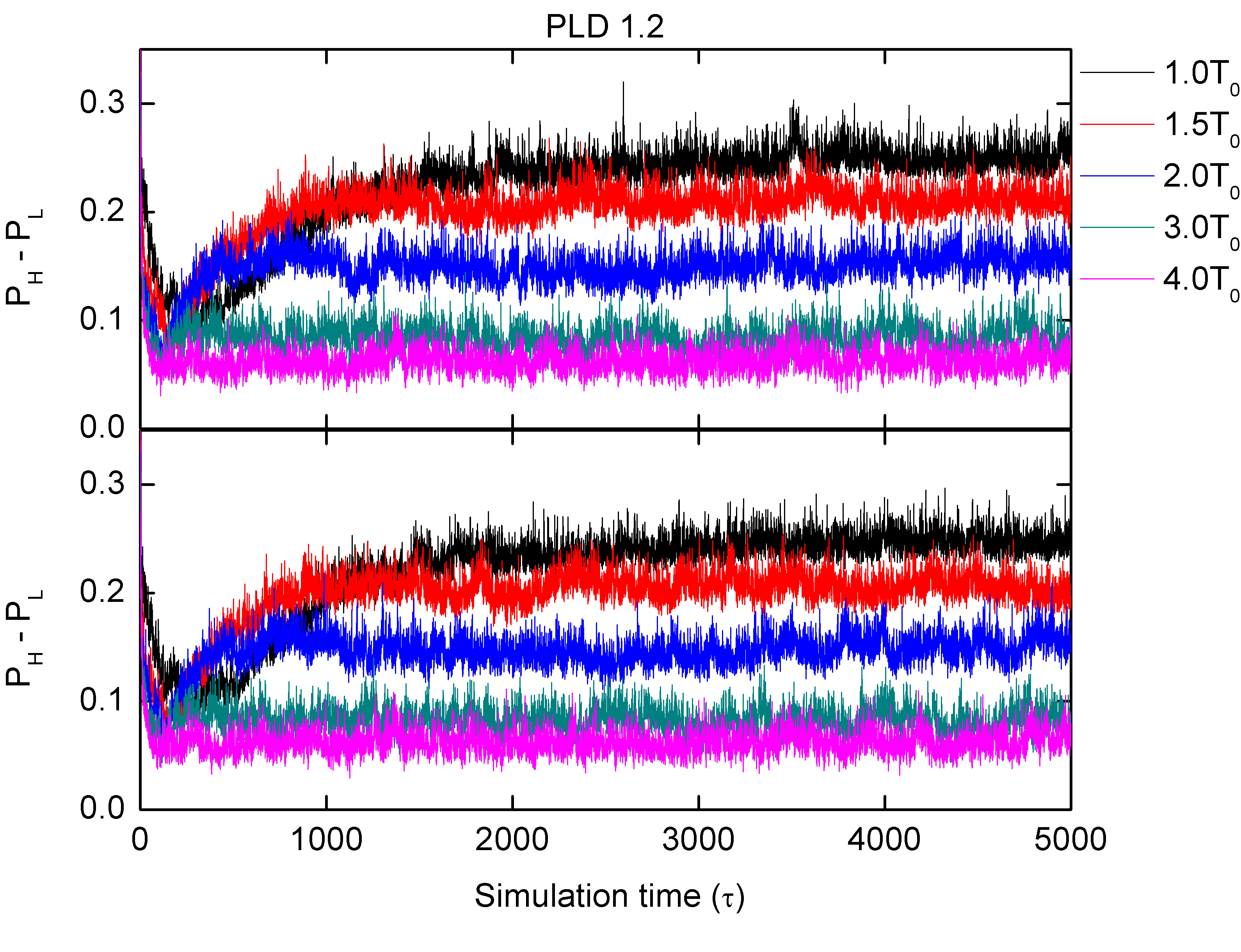

Supplement: Supplementary file 1 [file biomolecules-13-00625-s001.zip › FigS10.PLD1.2.png]

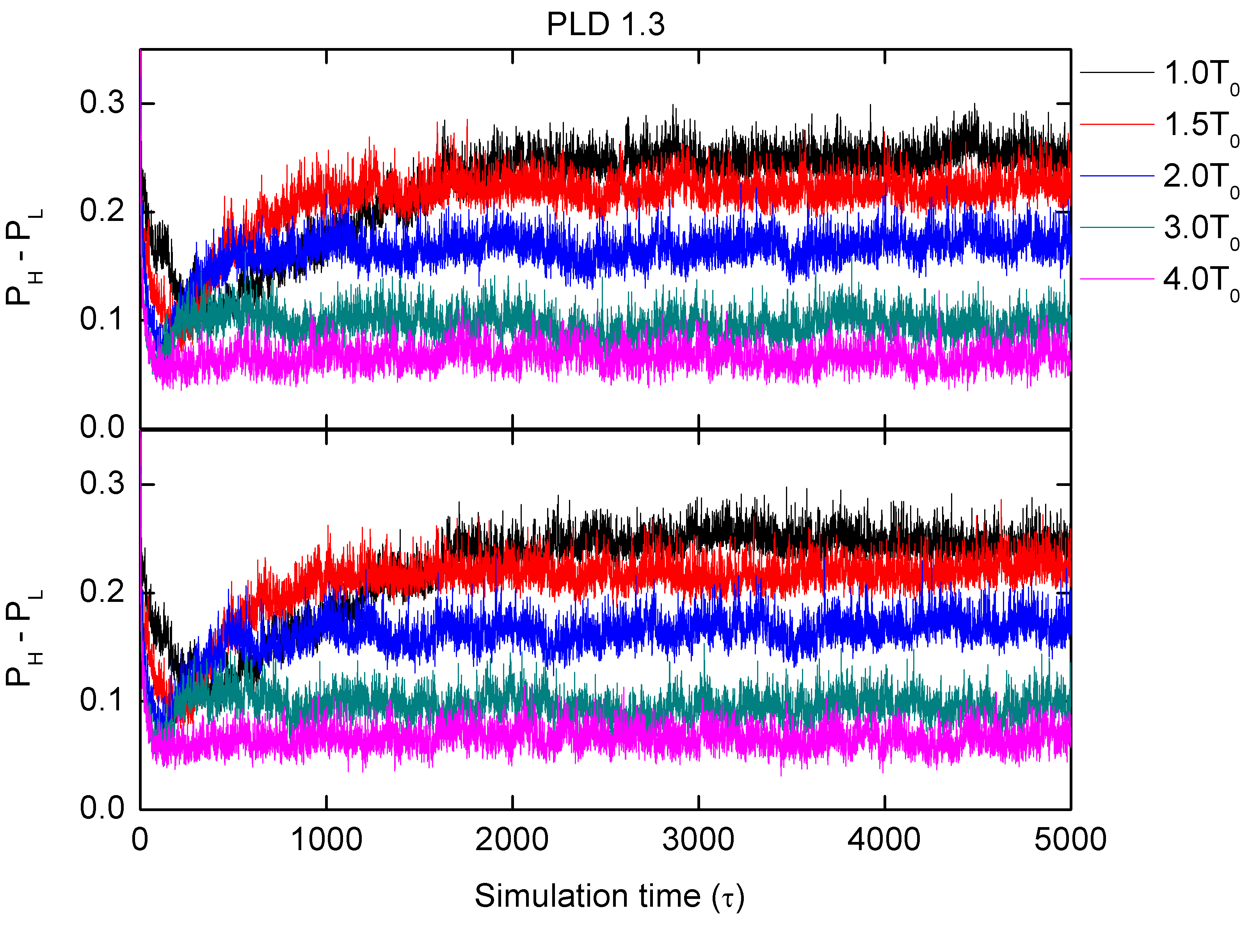

Supplement: Supplementary file 1 [file biomolecules-13-00625-s001.zip › FigS11.PLD1.3.png]

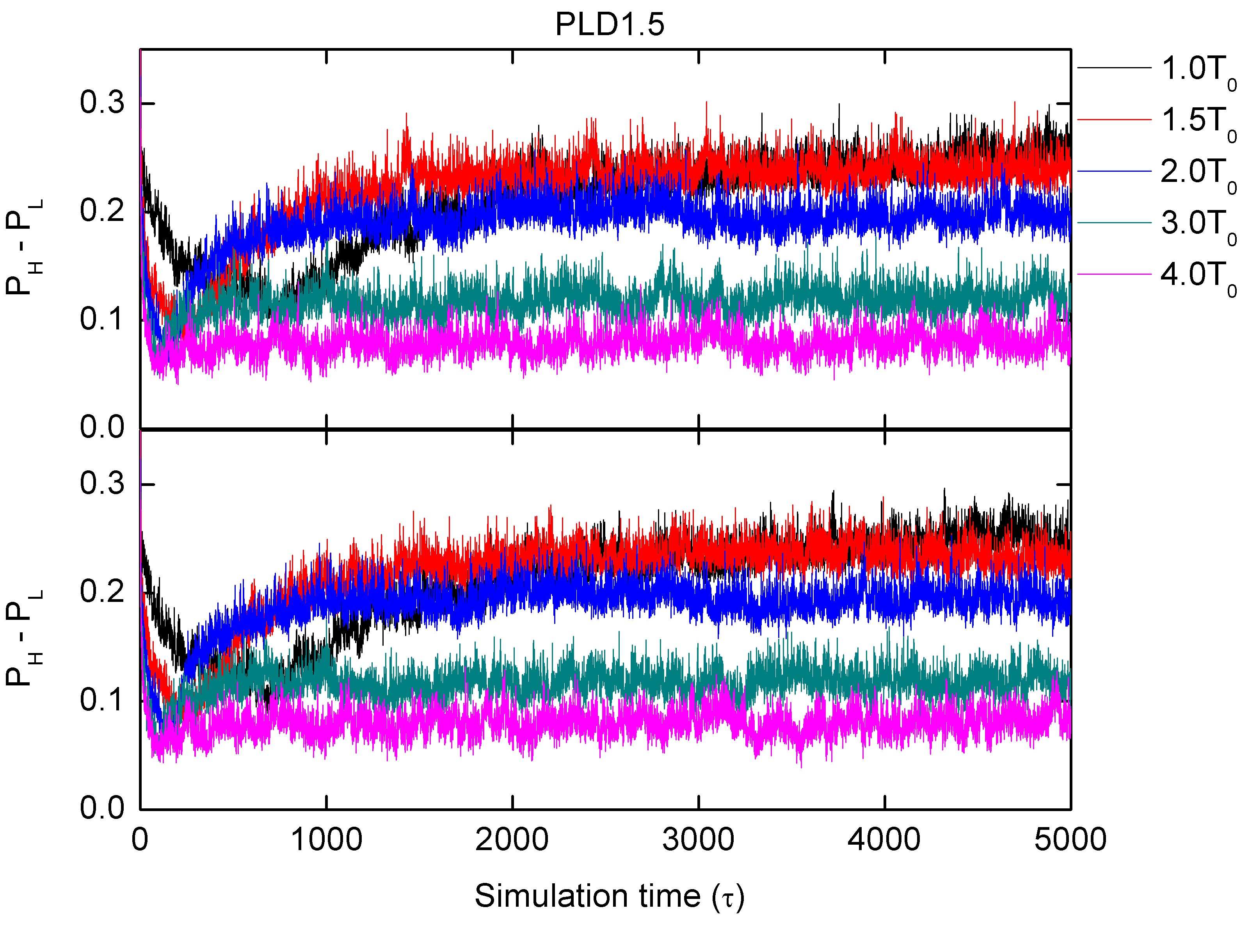

Supplement: Supplementary file 1 [file biomolecules-13-00625-s001.zip › FigS12.PLD1.5.png]

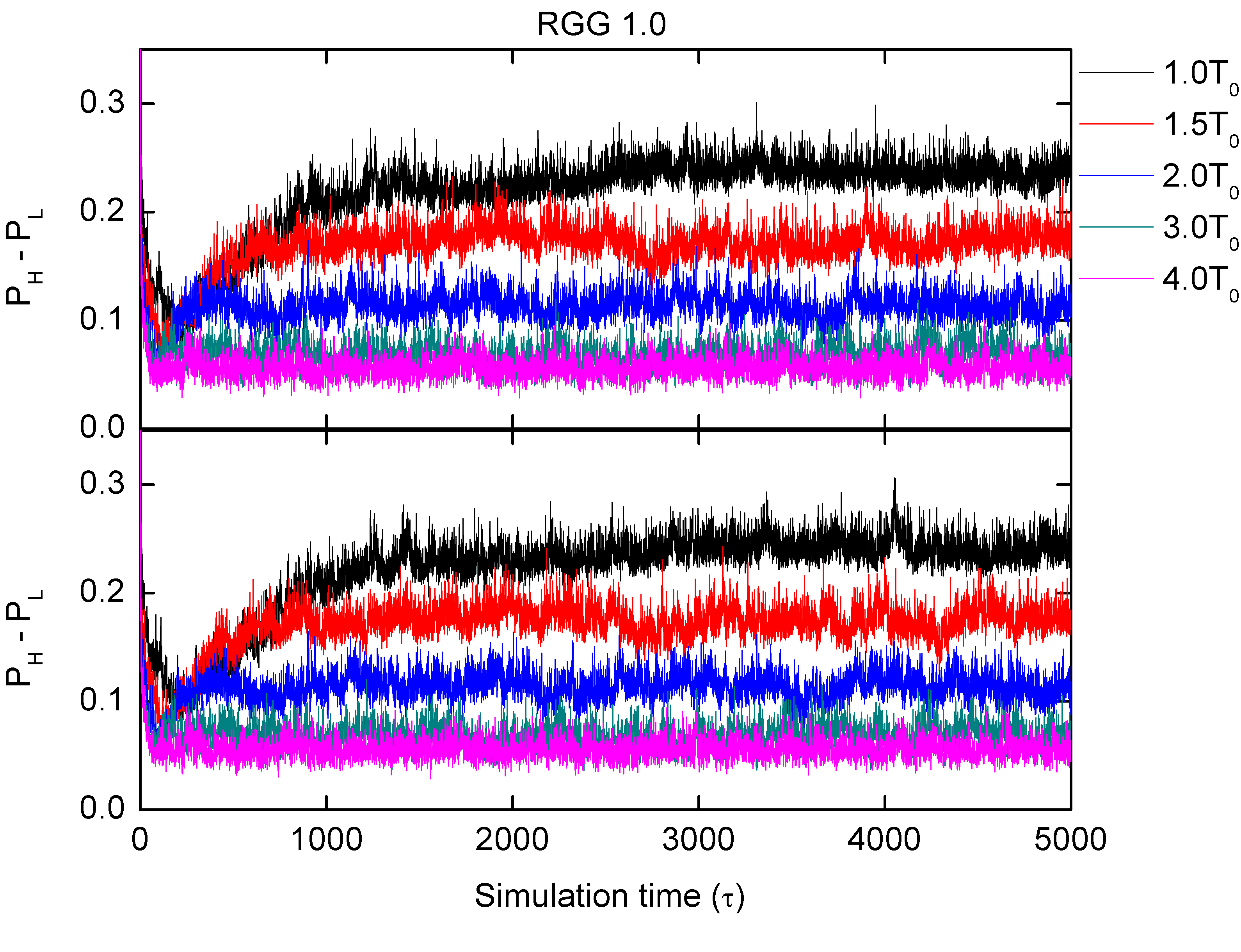

Supplement: Supplementary file 1 [file biomolecules-13-00625-s001.zip › FigS13.RGG1.0.png]

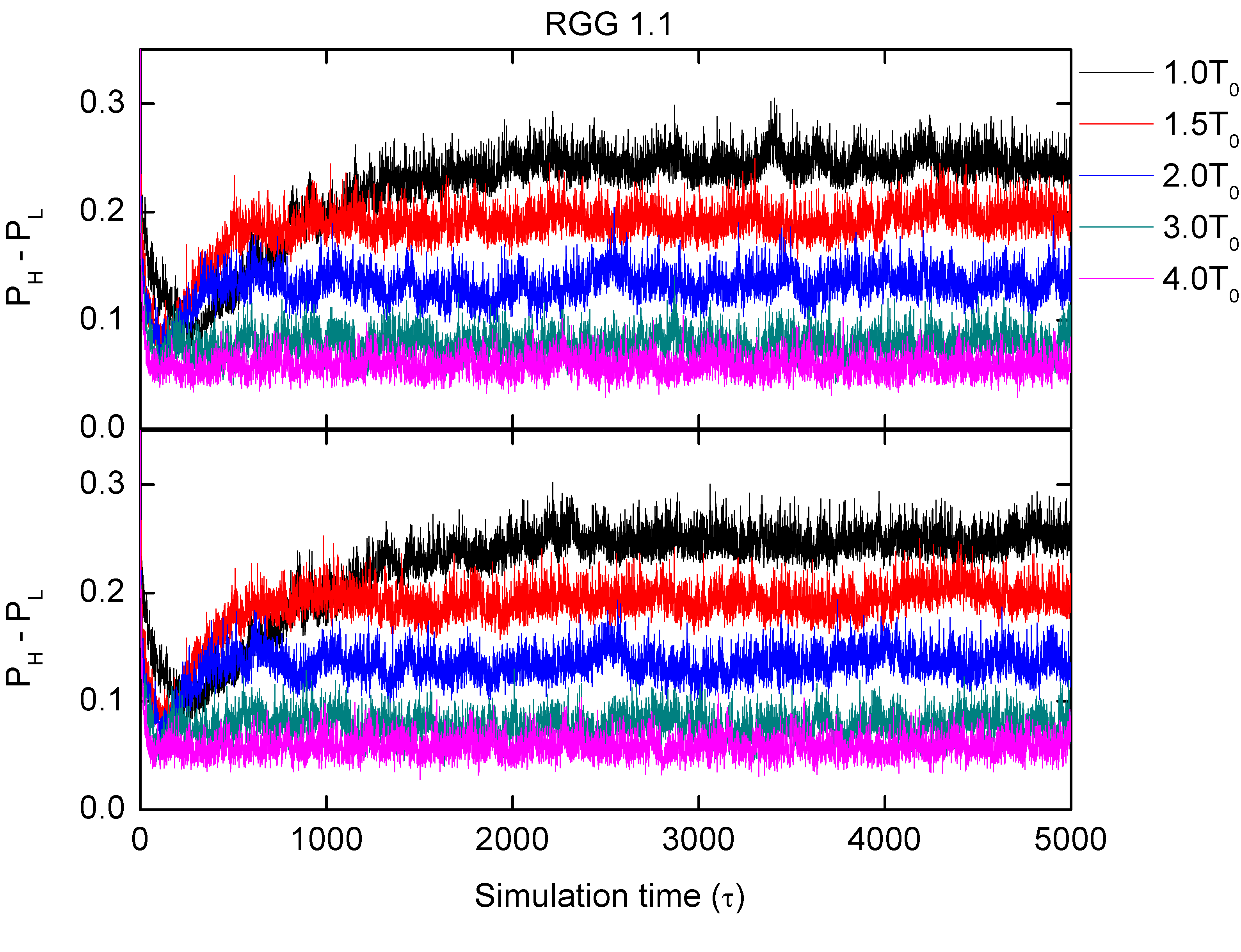

Supplement: Supplementary file 1 [file biomolecules-13-00625-s001.zip › FigS14.RGG1.1.png]

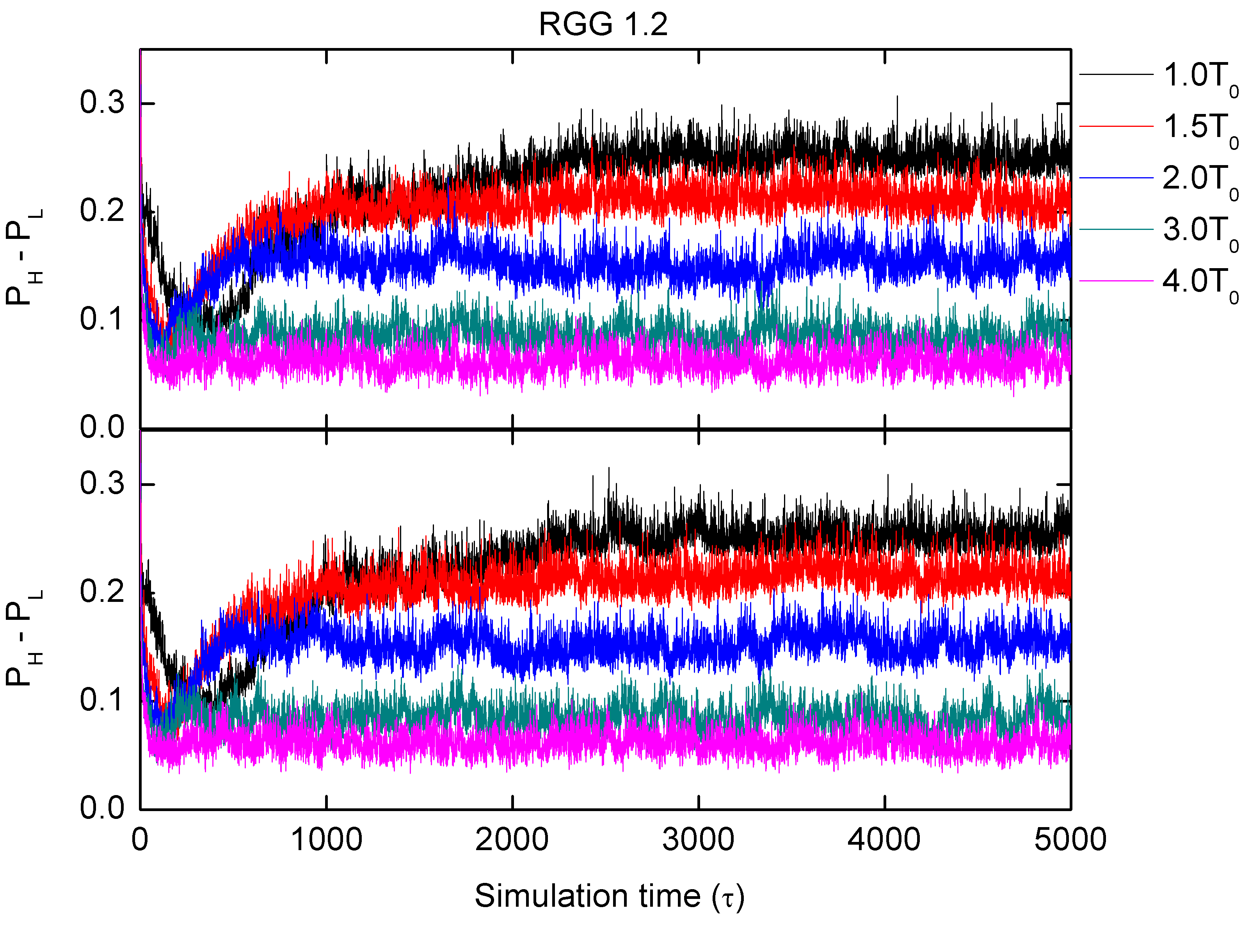

Supplement: Supplementary file 1 [file biomolecules-13-00625-s001.zip › FigS15.RGG1.2.png]

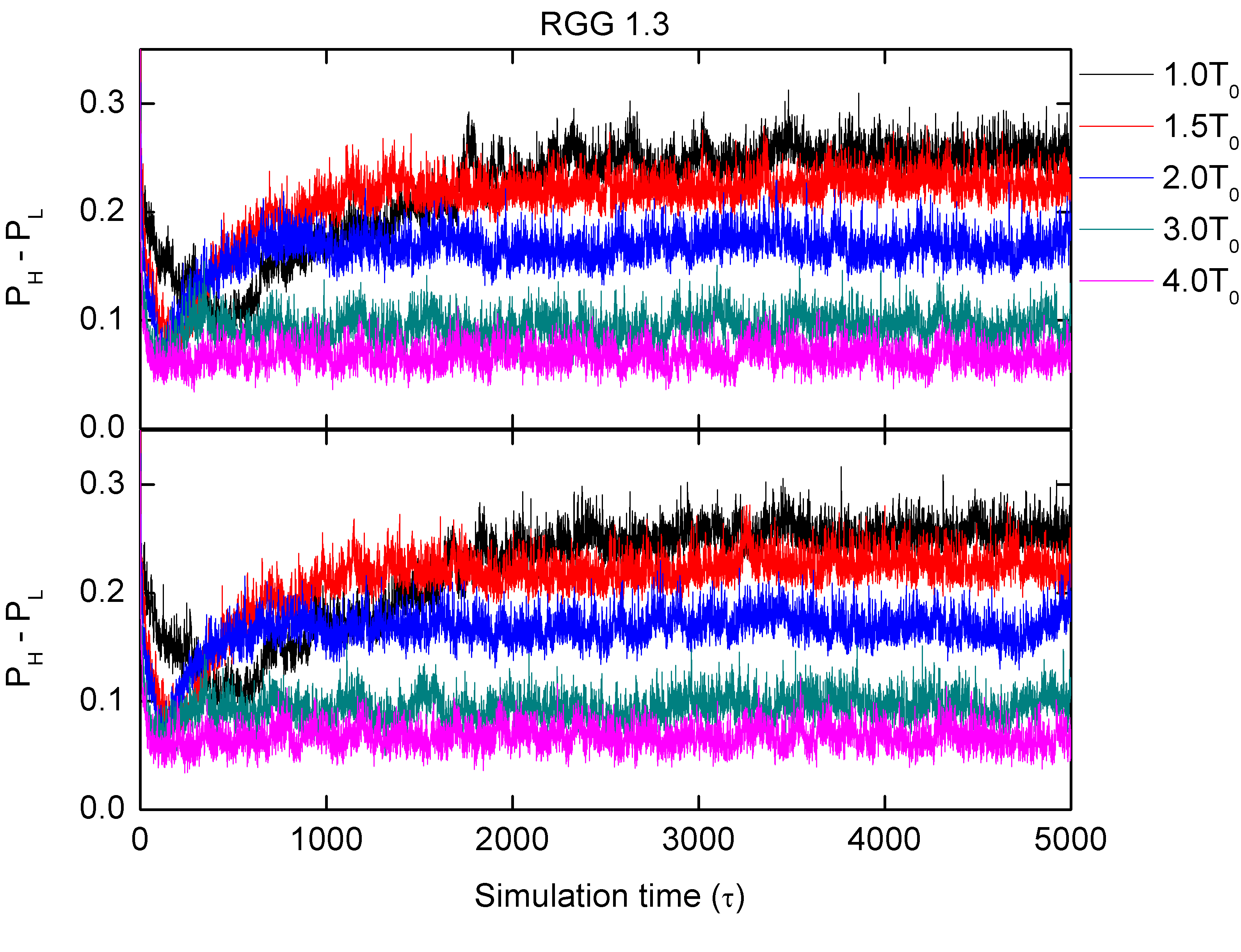

Supplement: Supplementary file 1 [file biomolecules-13-00625-s001.zip › FigS16.RGG1.3.png]

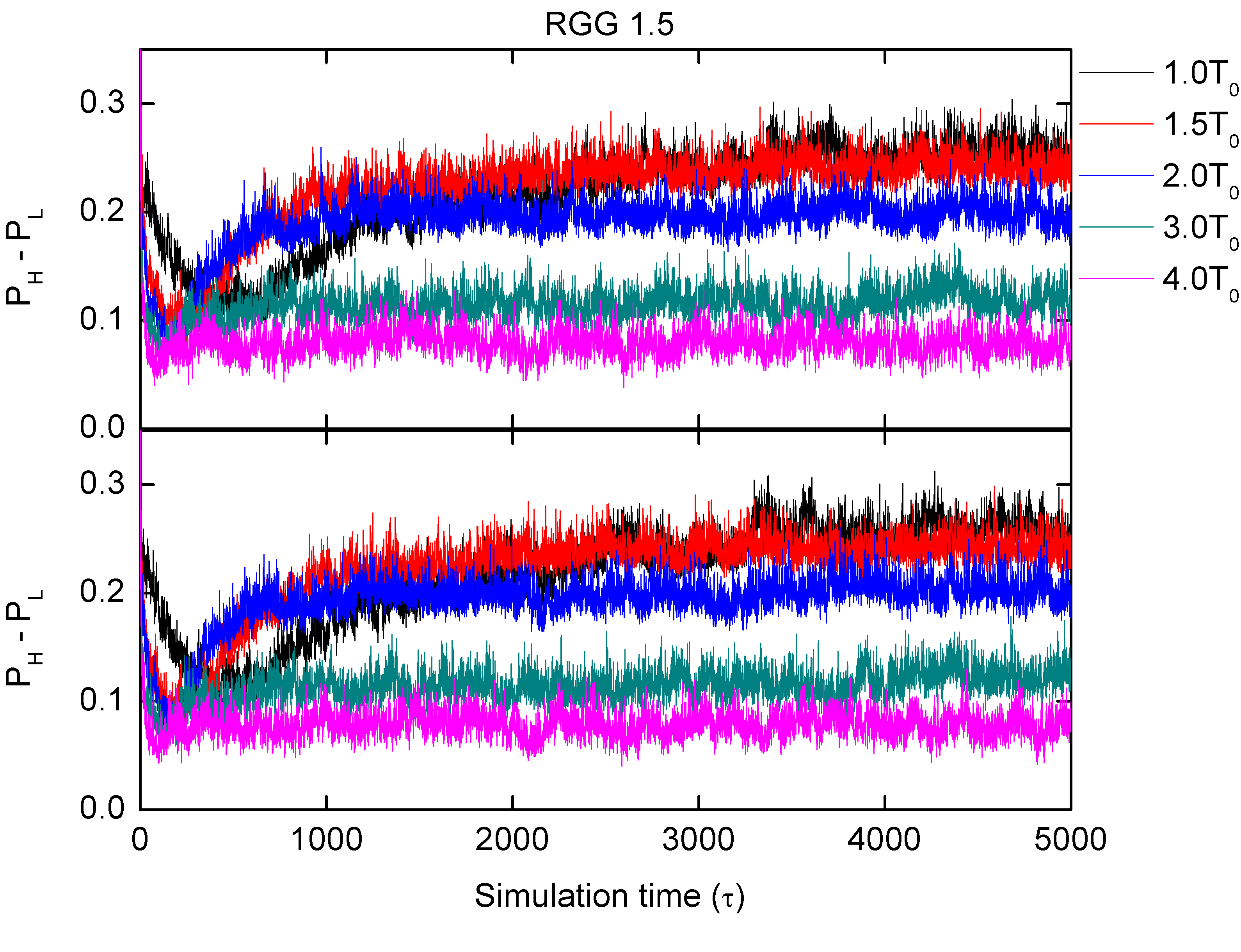

Supplement: Supplementary file 1 [file biomolecules-13-00625-s001.zip › FigS17.RGG1.5.png]

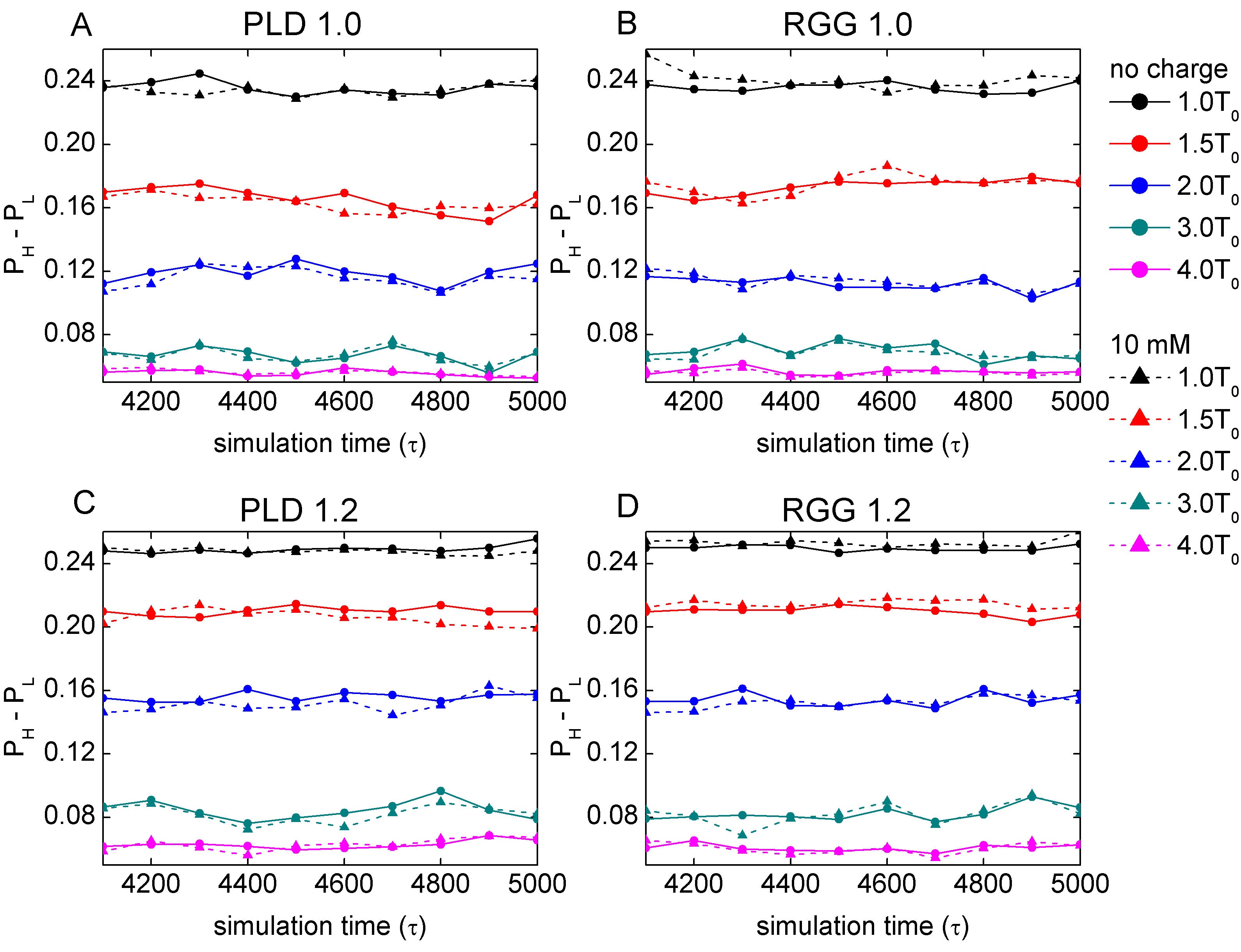

Supplement: Supplementary file 1 [file biomolecules-13-00625-s001.zip › FigS18.pr_t100-PH-PL.png]

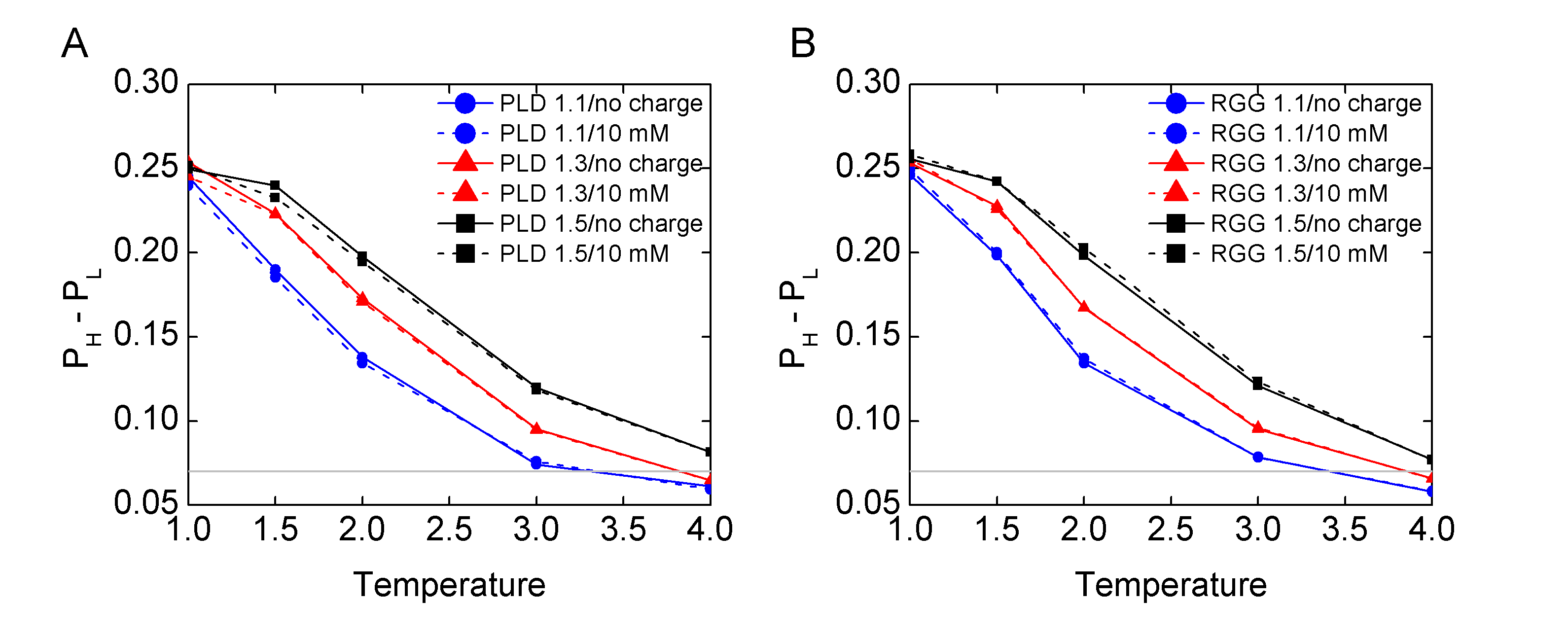

Supplement: Supplementary file 1 [file biomolecules-13-00625-s001.zip › FigS19.prT_PH-PL.png]

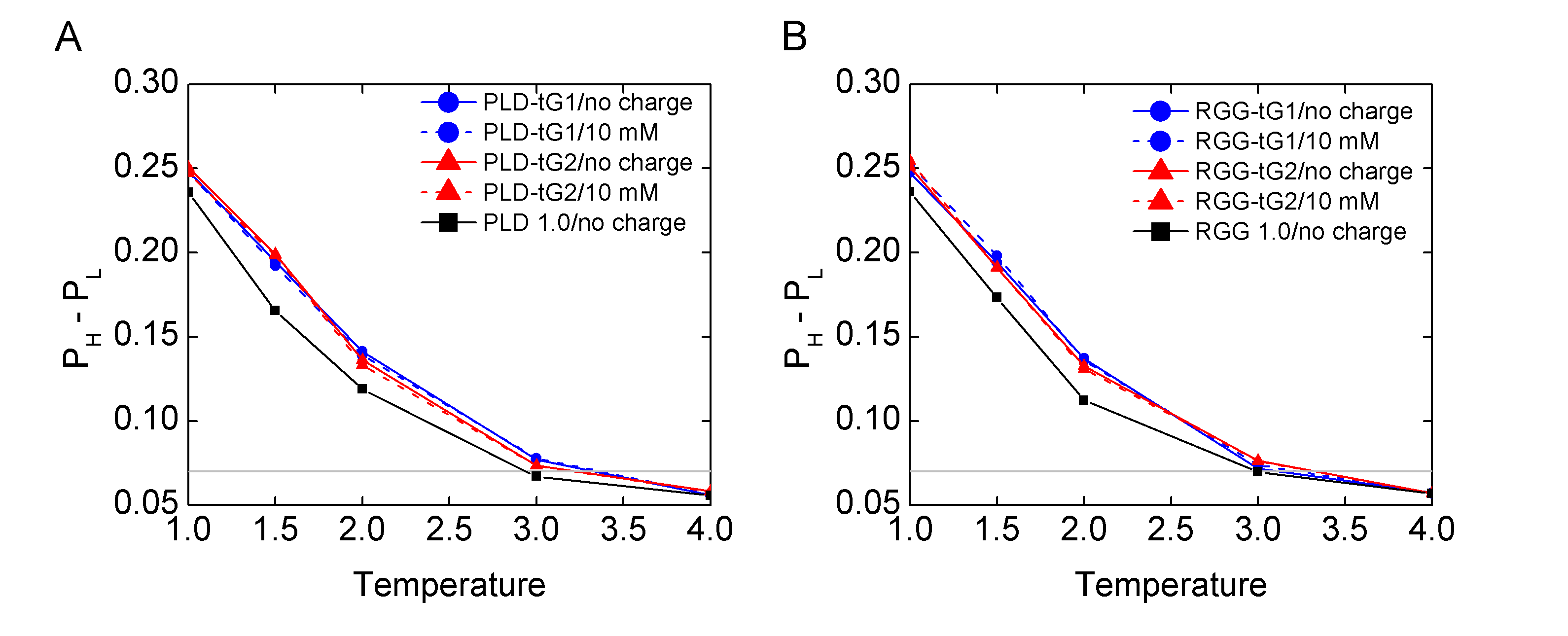

Supplement: Supplementary file 1 [file biomolecules-13-00625-s001.zip › FigS2.tGPH-PL(epsilon1).png]

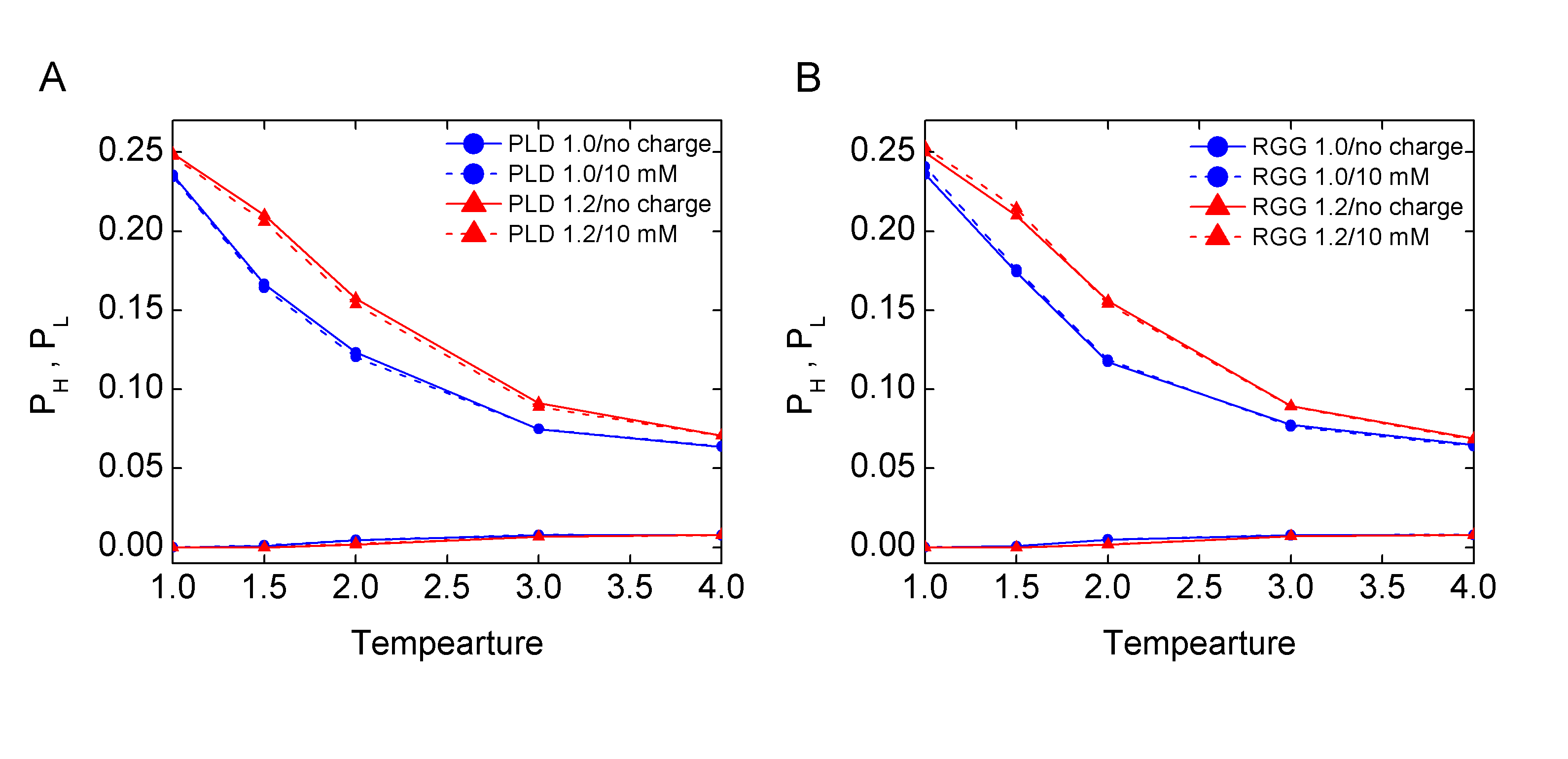

Supplement: Supplementary file 1 [file biomolecules-13-00625-s001.zip › FigS20.PH&PL.png]

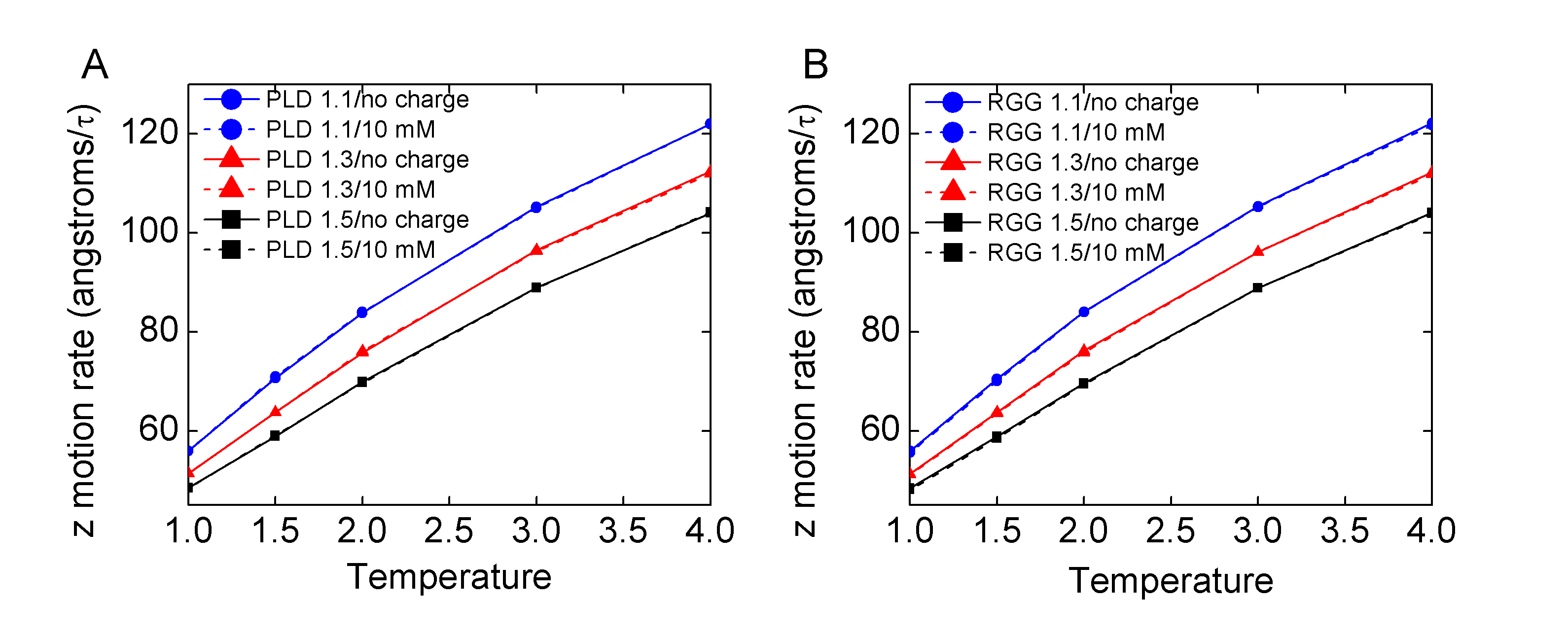

Supplement: Supplementary file 1 [file biomolecules-13-00625-s001.zip › FigS21.pr6_Tvelcom.png]

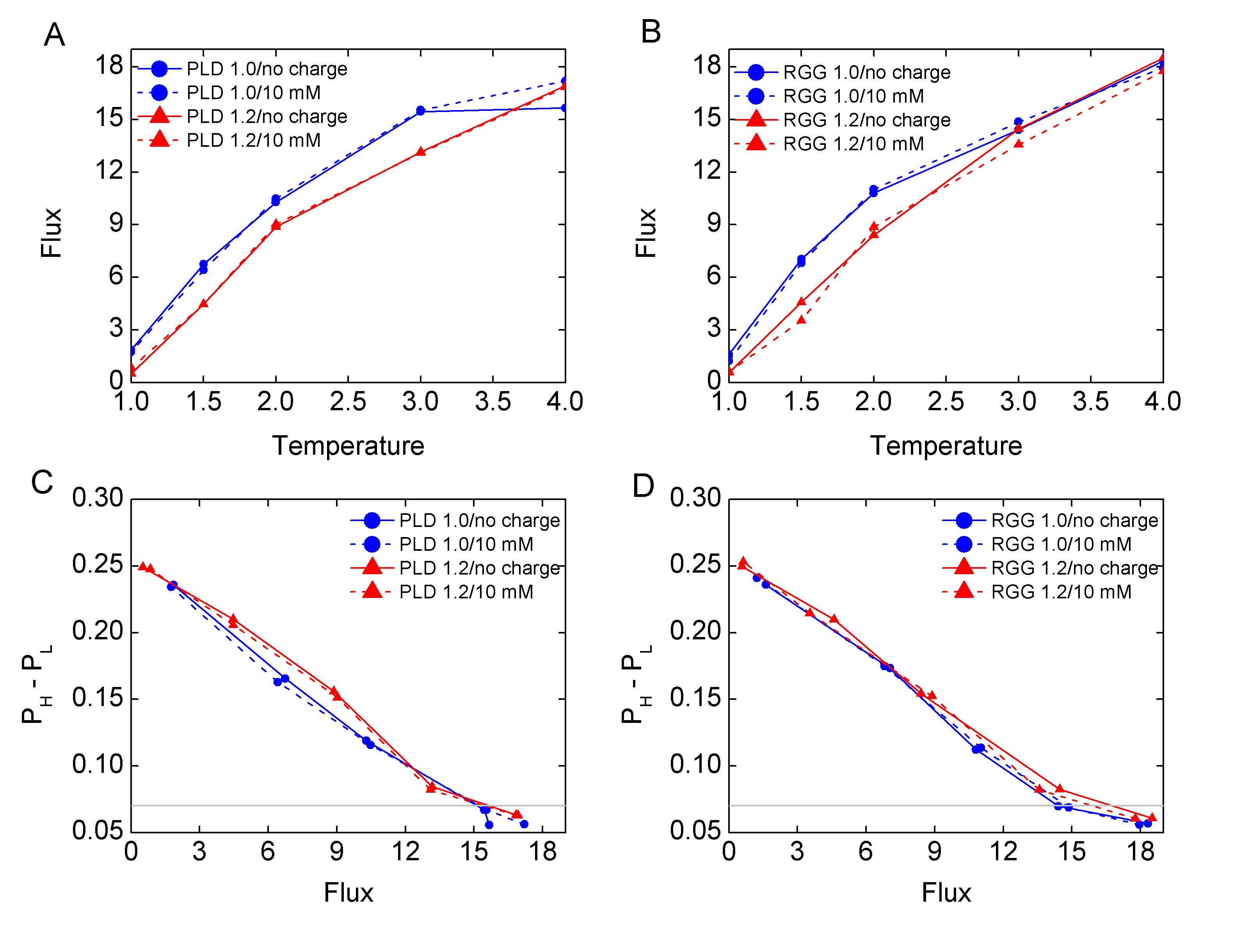

Supplement: Supplementary file 1 [file biomolecules-13-00625-s001.zip › FigS22.t100-T-mExch.png]

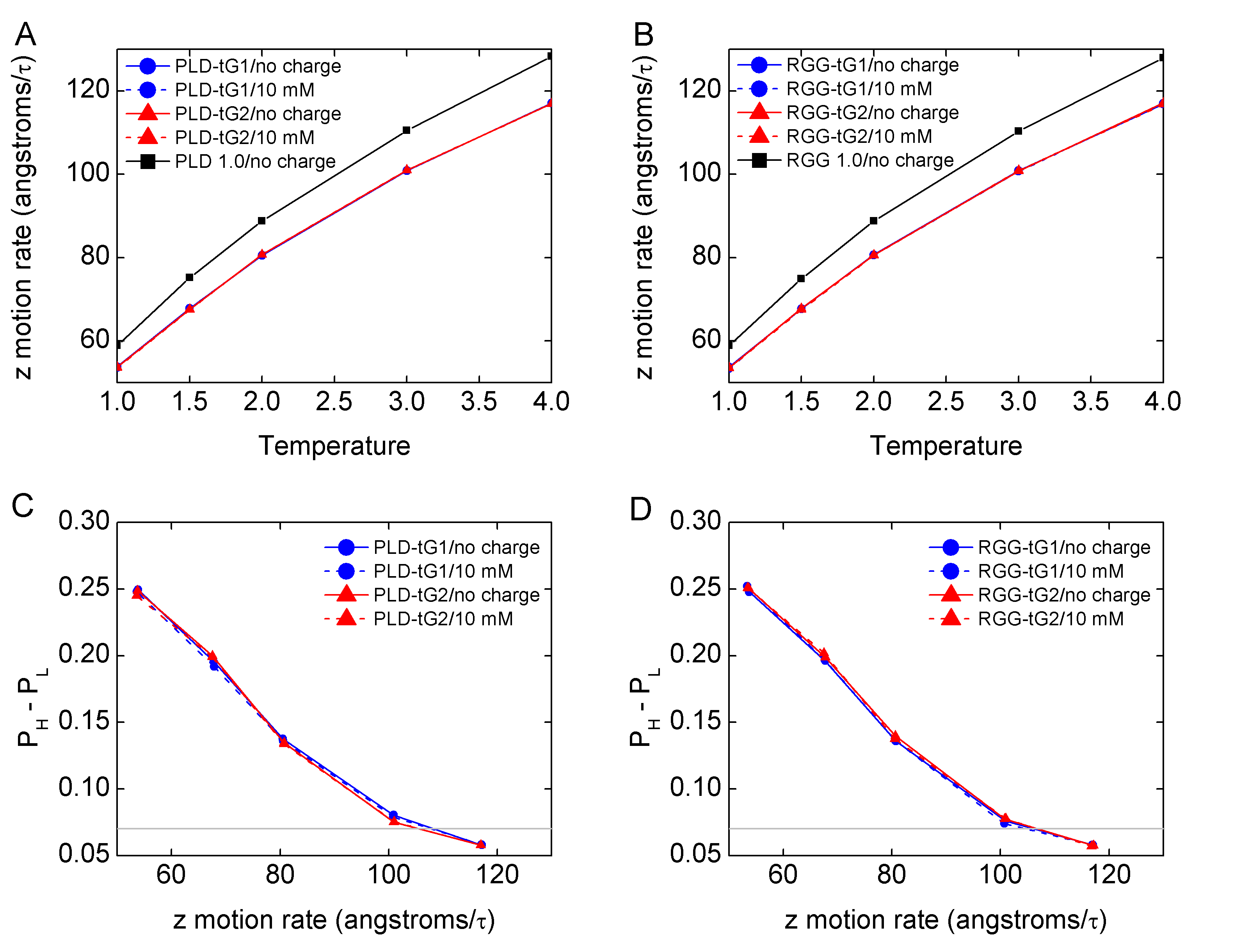

Supplement: Supplementary file 1 [file biomolecules-13-00625-s001.zip › FigS23.tGT-mvelcom_4.png]

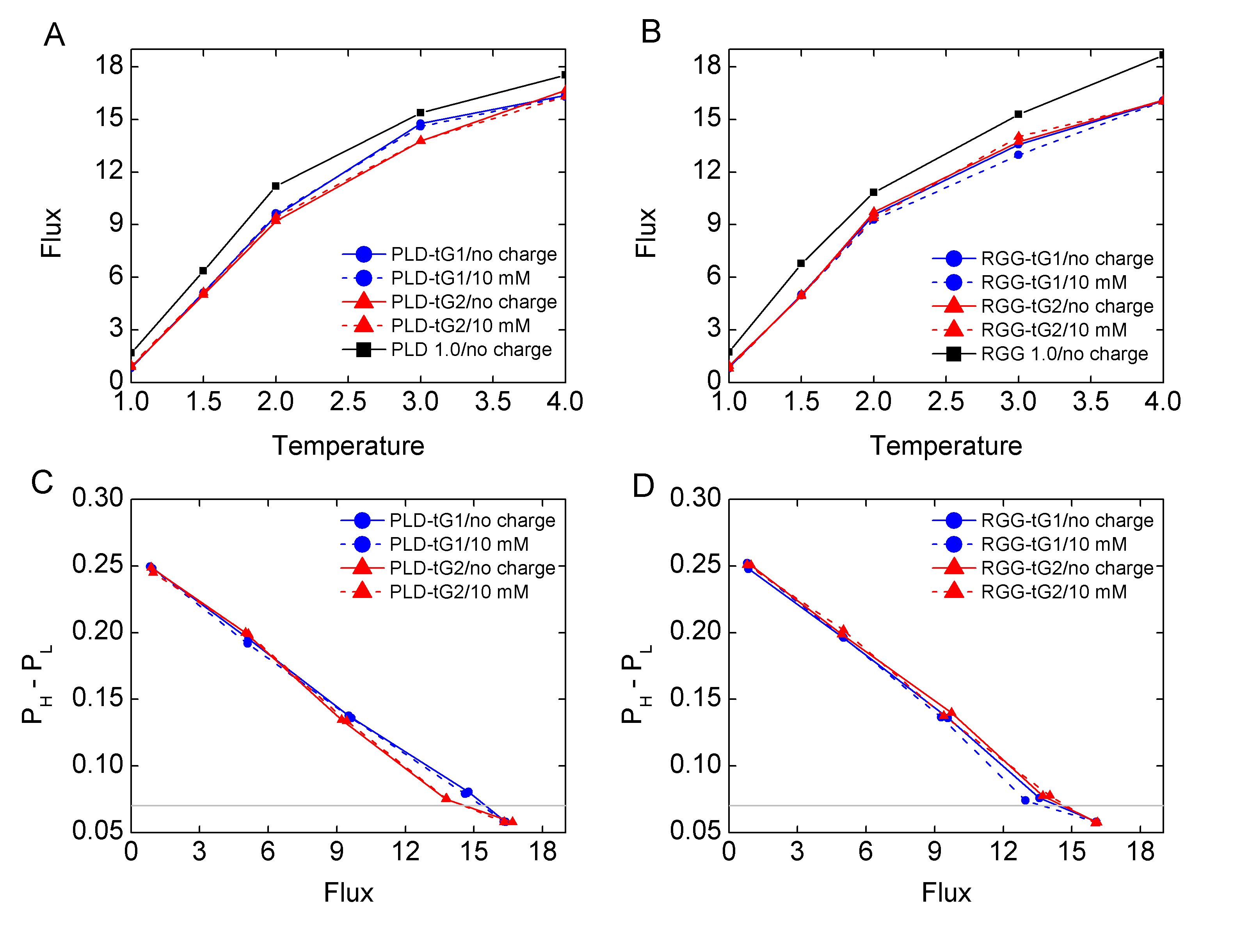

Supplement: Supplementary file 1 [file biomolecules-13-00625-s001.zip › FigS24.tGT-mExch.png]

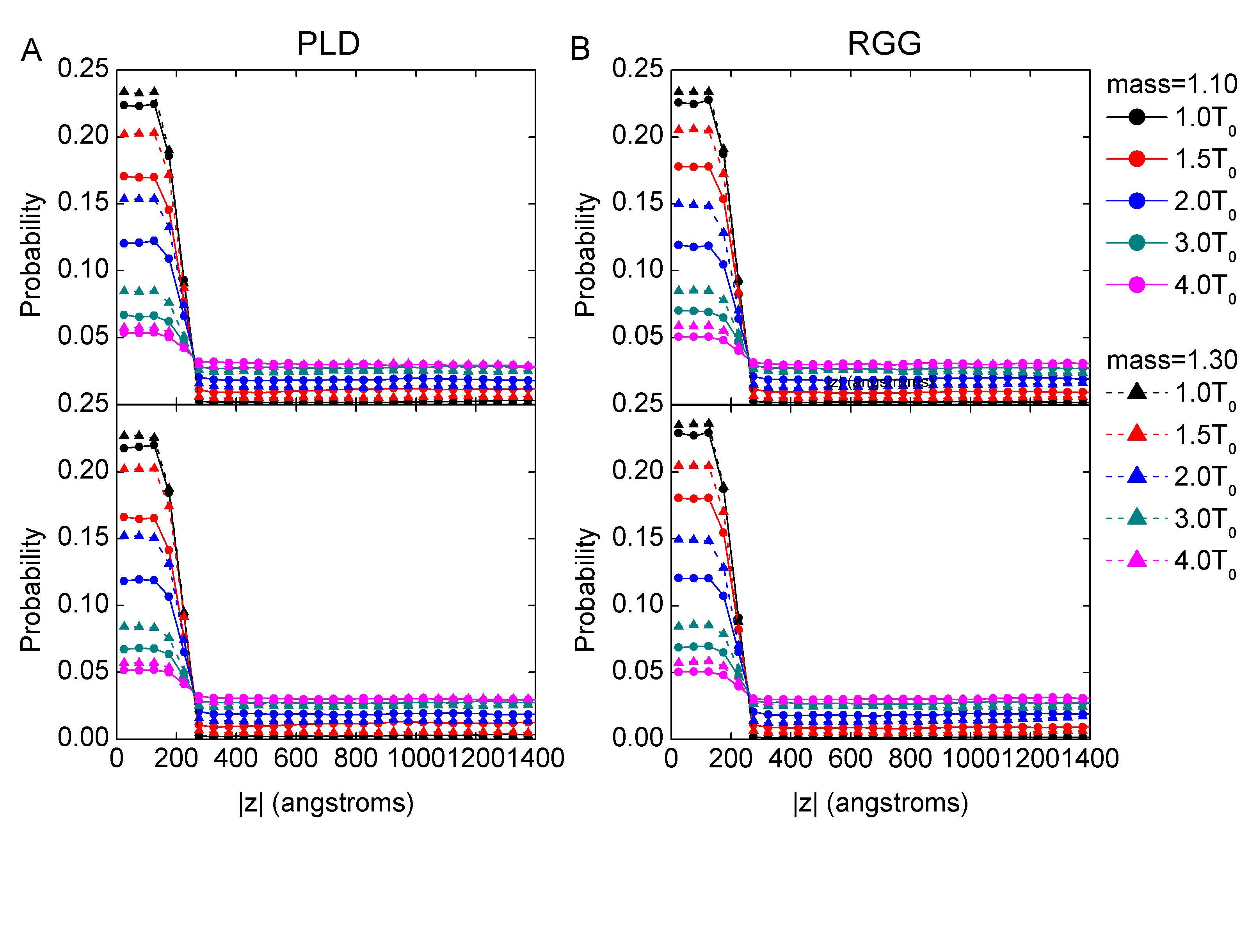

Supplement: Supplementary file 1 [file biomolecules-13-00625-s001.zip › FigS25.pr1113_wPchain.png]

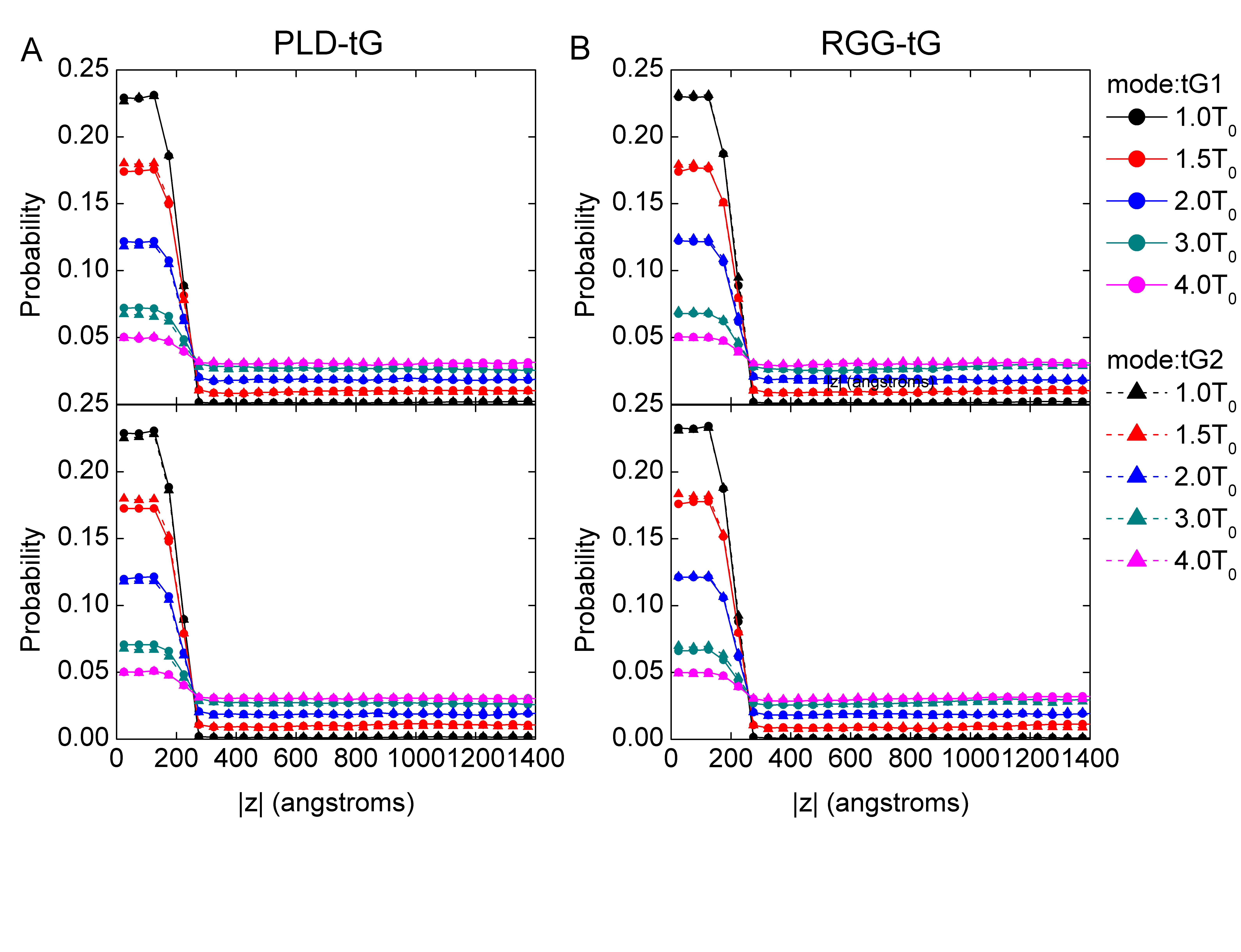

Supplement: Supplementary file 1 [file biomolecules-13-00625-s001.zip › FigS26.tGProbability.png]

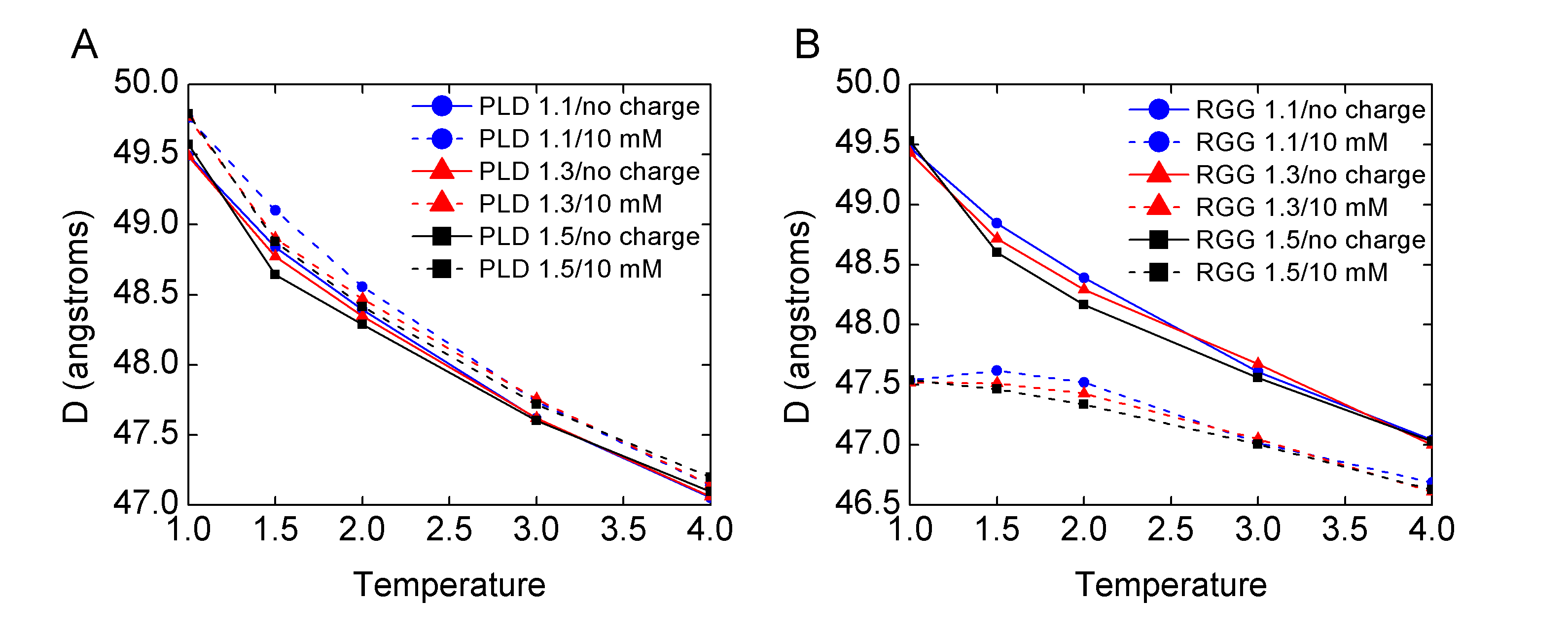

Supplement: Supplementary file 1 [file biomolecules-13-00625-s001.zip › FigS27.pr6_TmD.png]

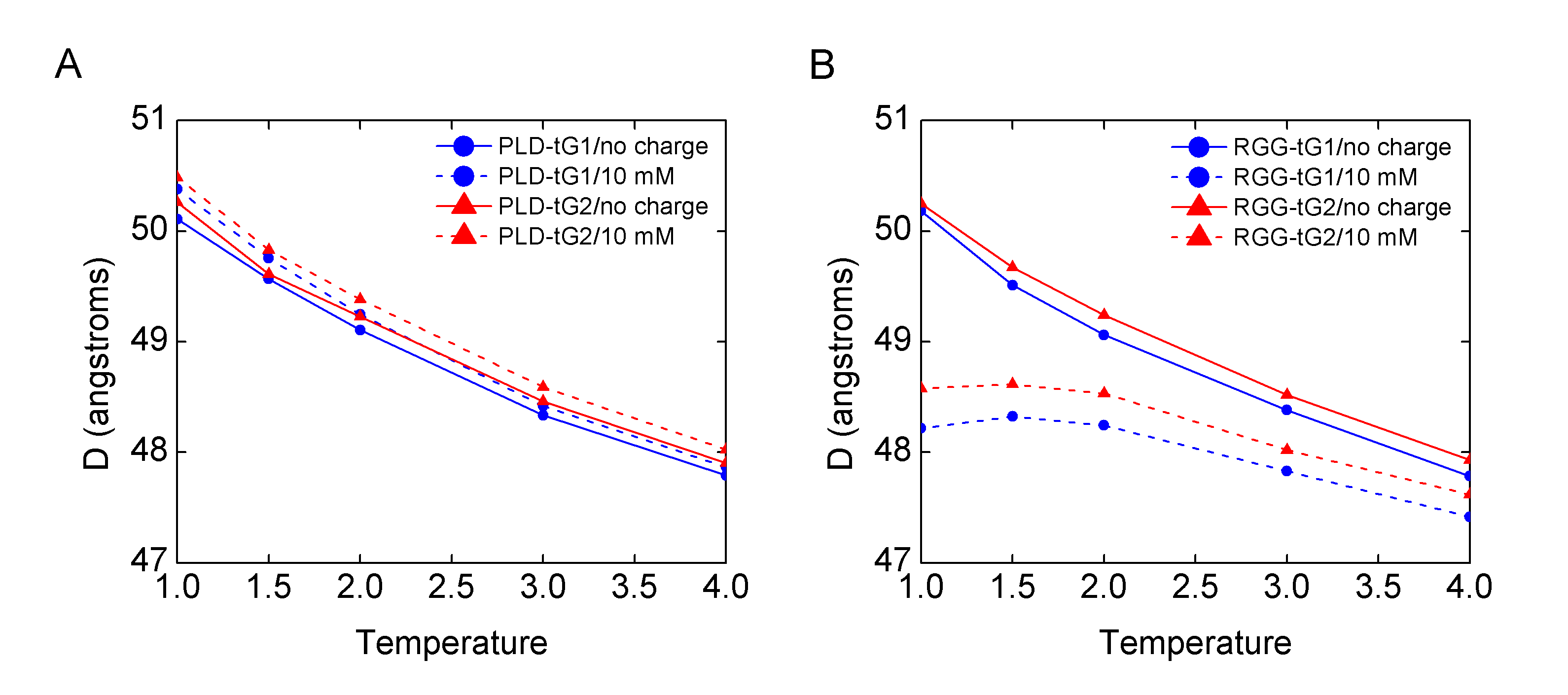

Supplement: Supplementary file 1 [file biomolecules-13-00625-s001.zip › FigS28.tGT-mD2.png]

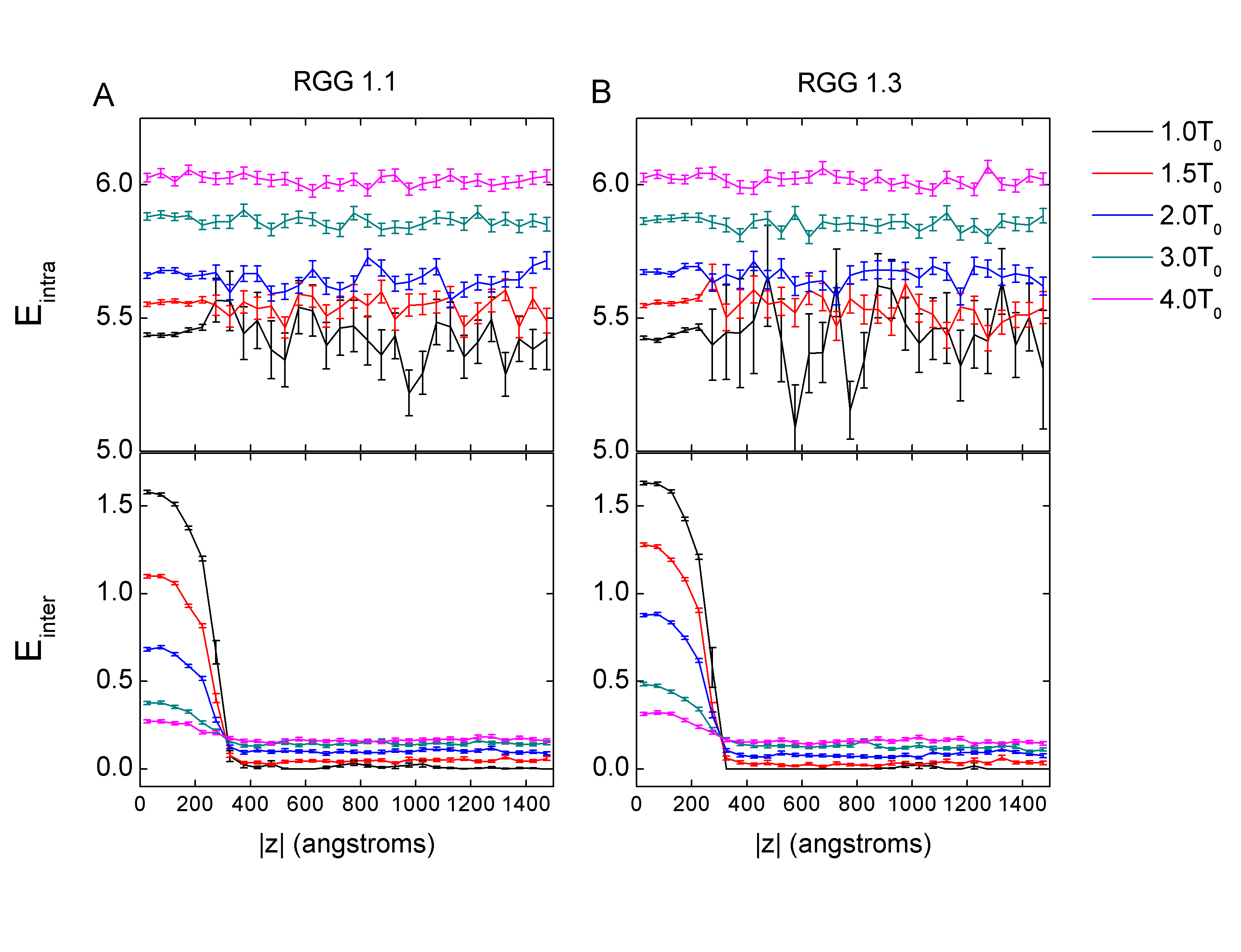

Supplement: Supplementary file 1 [file biomolecules-13-00625-s001.zip › FigS29.Eintra_inter_4.png]

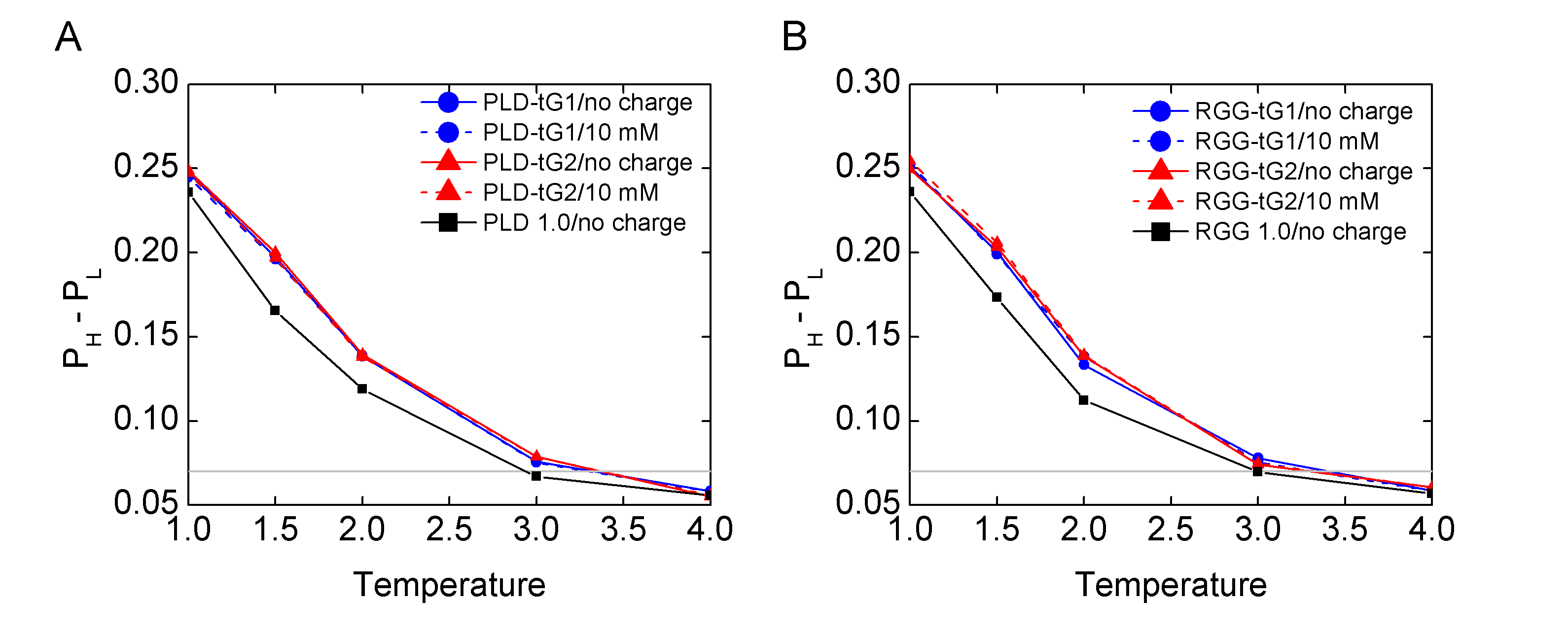

Supplement: Supplementary file 1 [file biomolecules-13-00625-s001.zip › FigS3.tGPH-PL(epsilon3).png]

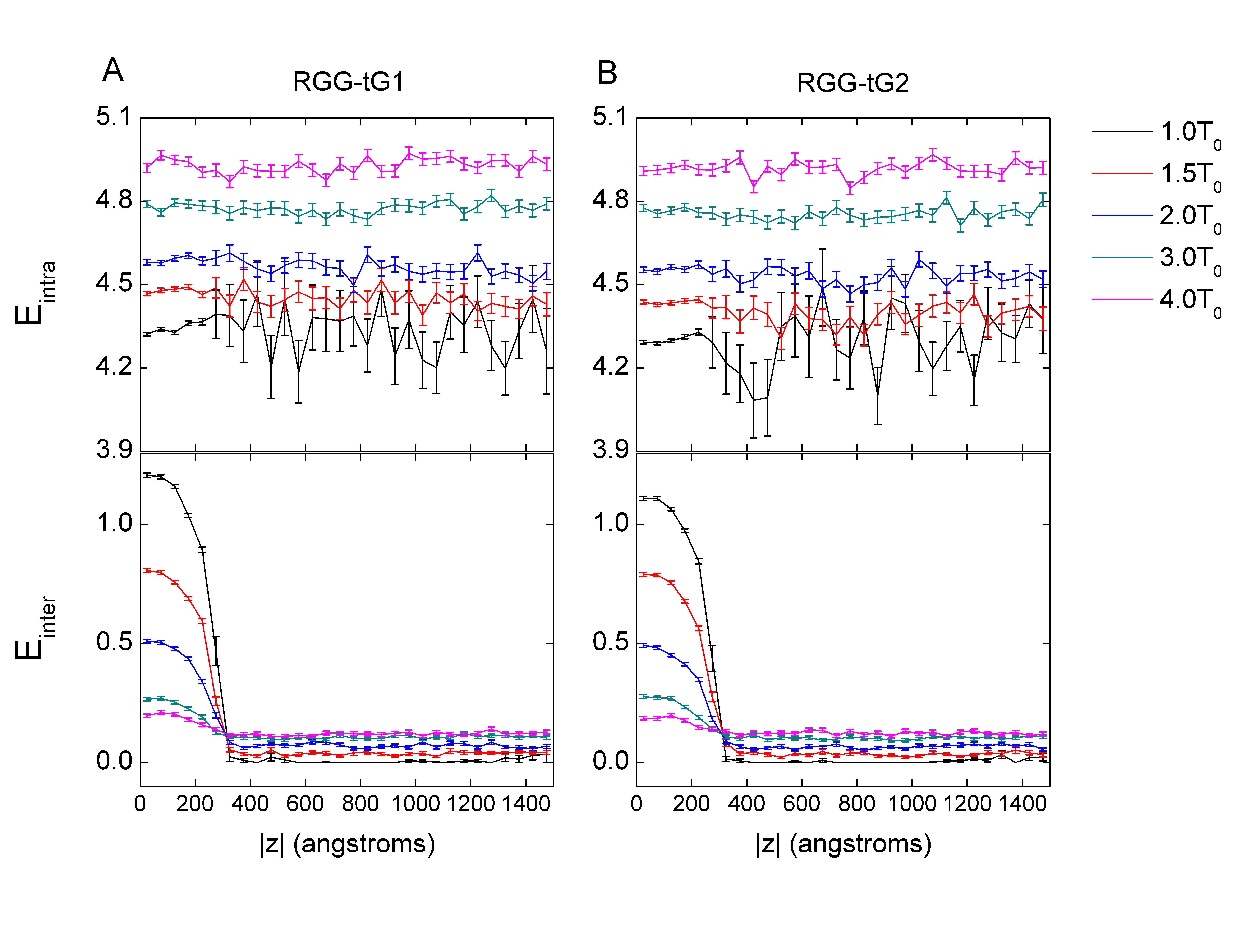

Supplement: Supplementary file 1 [file biomolecules-13-00625-s001.zip › FigS30.tGEintra_inter.png]

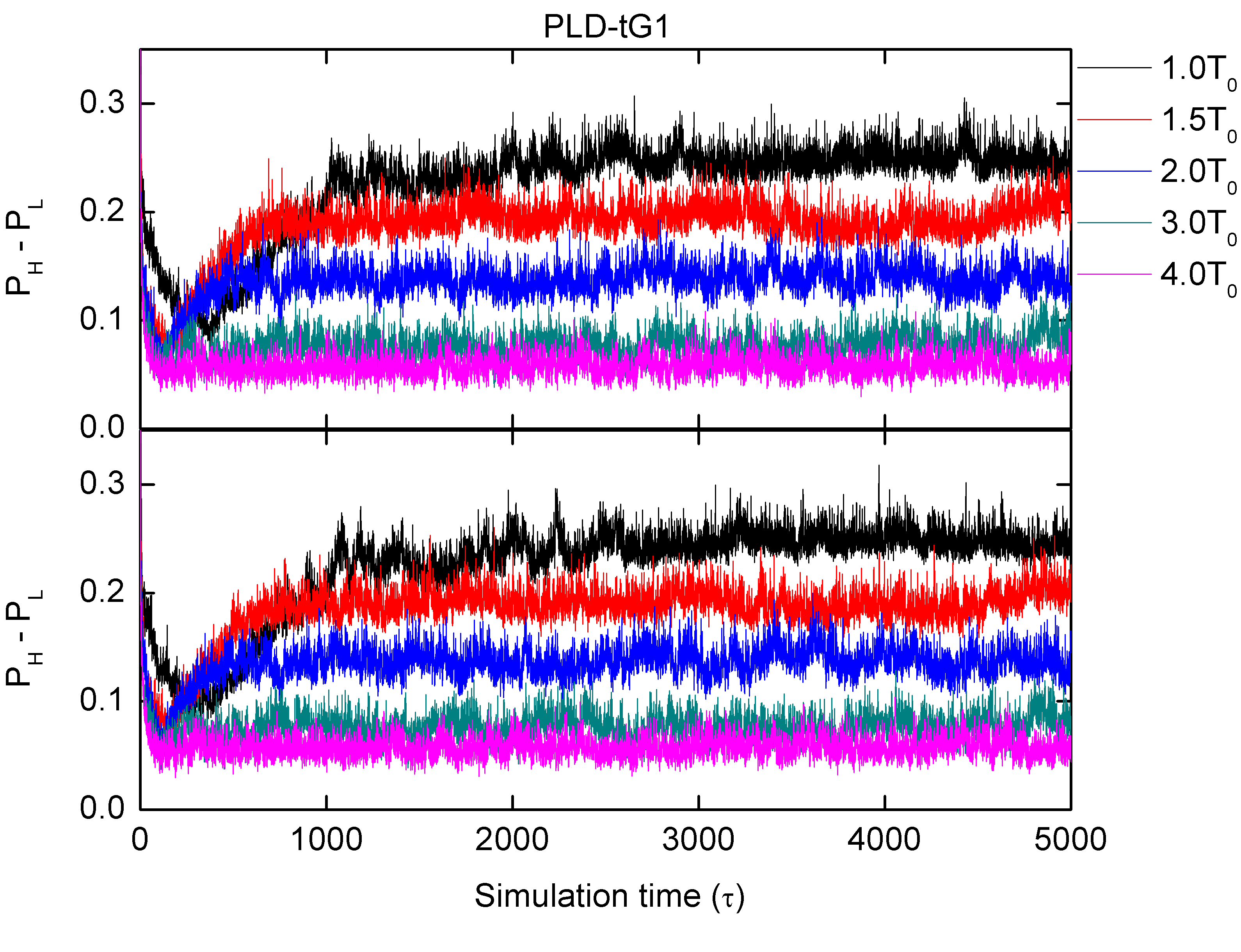

Supplement: Supplementary file 1 [file biomolecules-13-00625-s001.zip › FigS4.PLD-tG1.png]

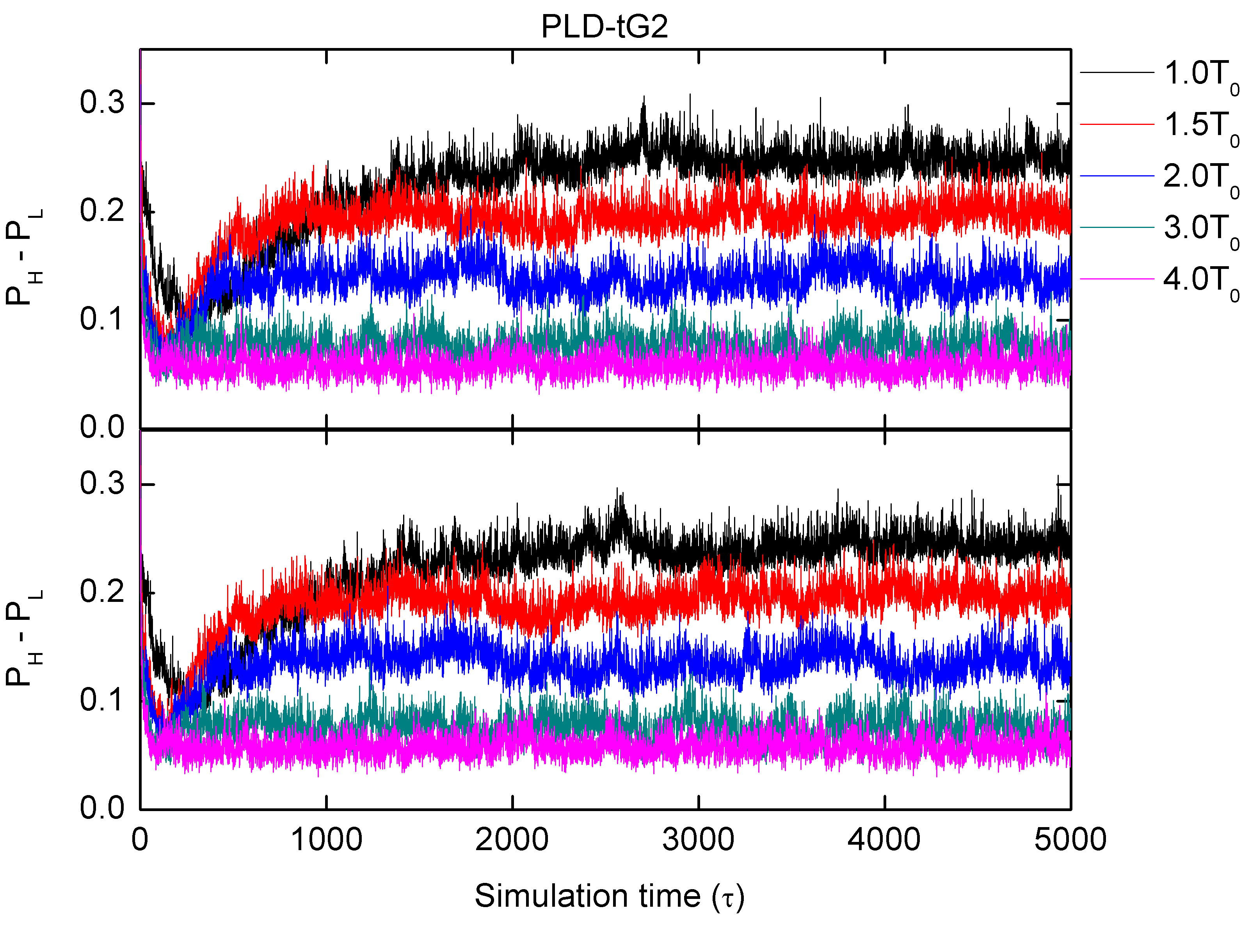

Supplement: Supplementary file 1 [file biomolecules-13-00625-s001.zip › FigS5.PLD-tG2.png]

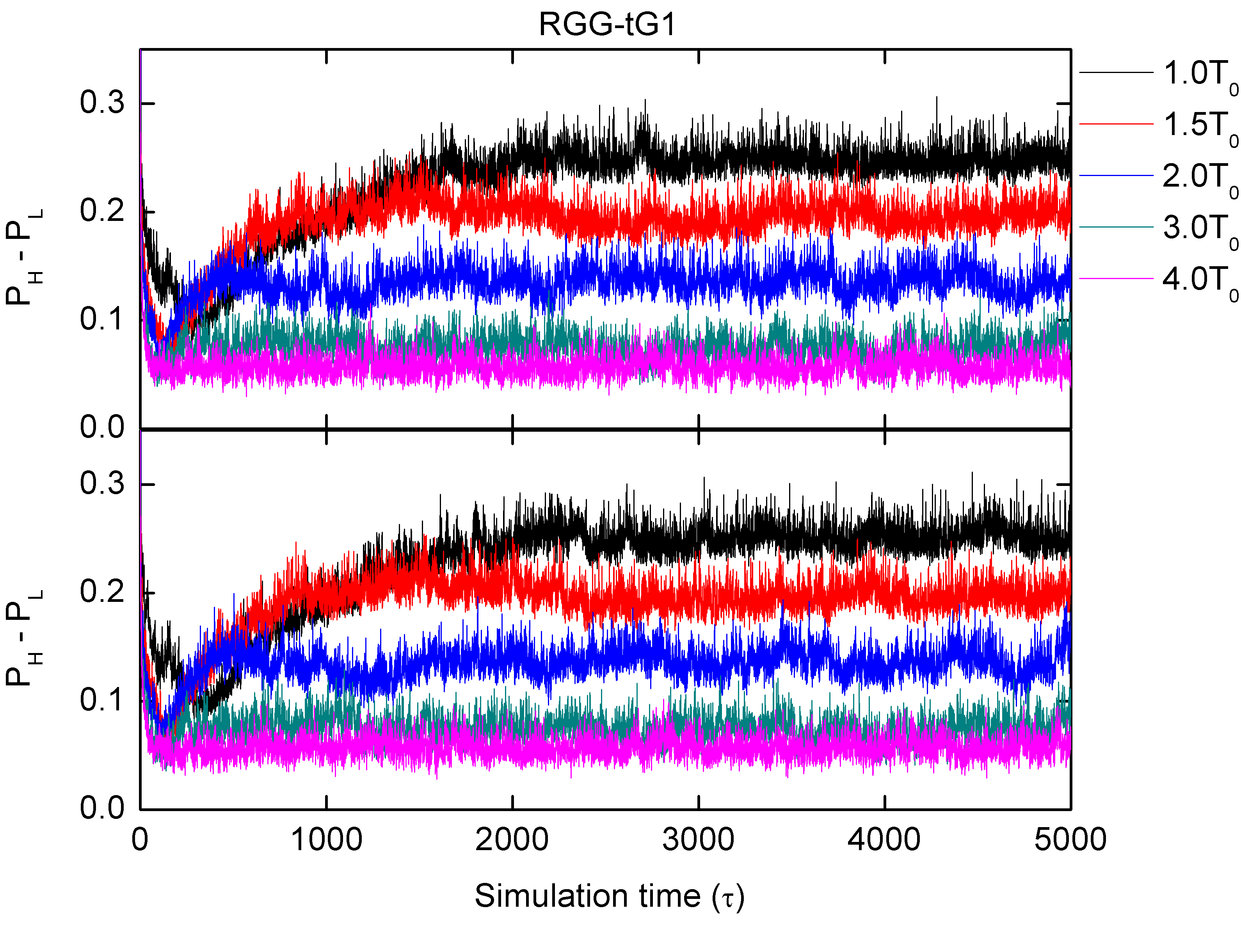

Supplement: Supplementary file 1 [file biomolecules-13-00625-s001.zip › FigS6.RGG-tG1.png]

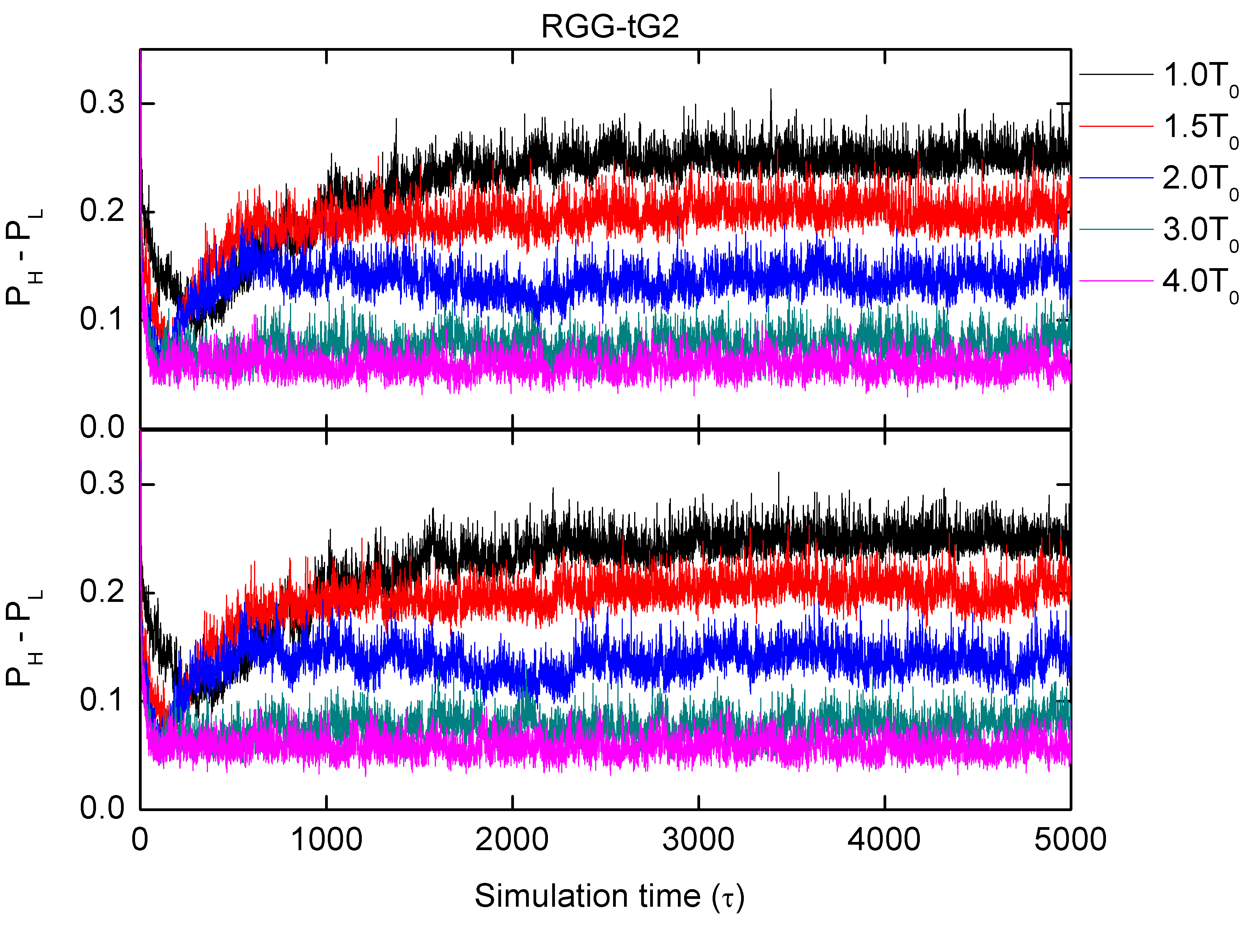

Supplement: Supplementary file 1 [file biomolecules-13-00625-s001.zip › FigS7.RGG-tG2.png]

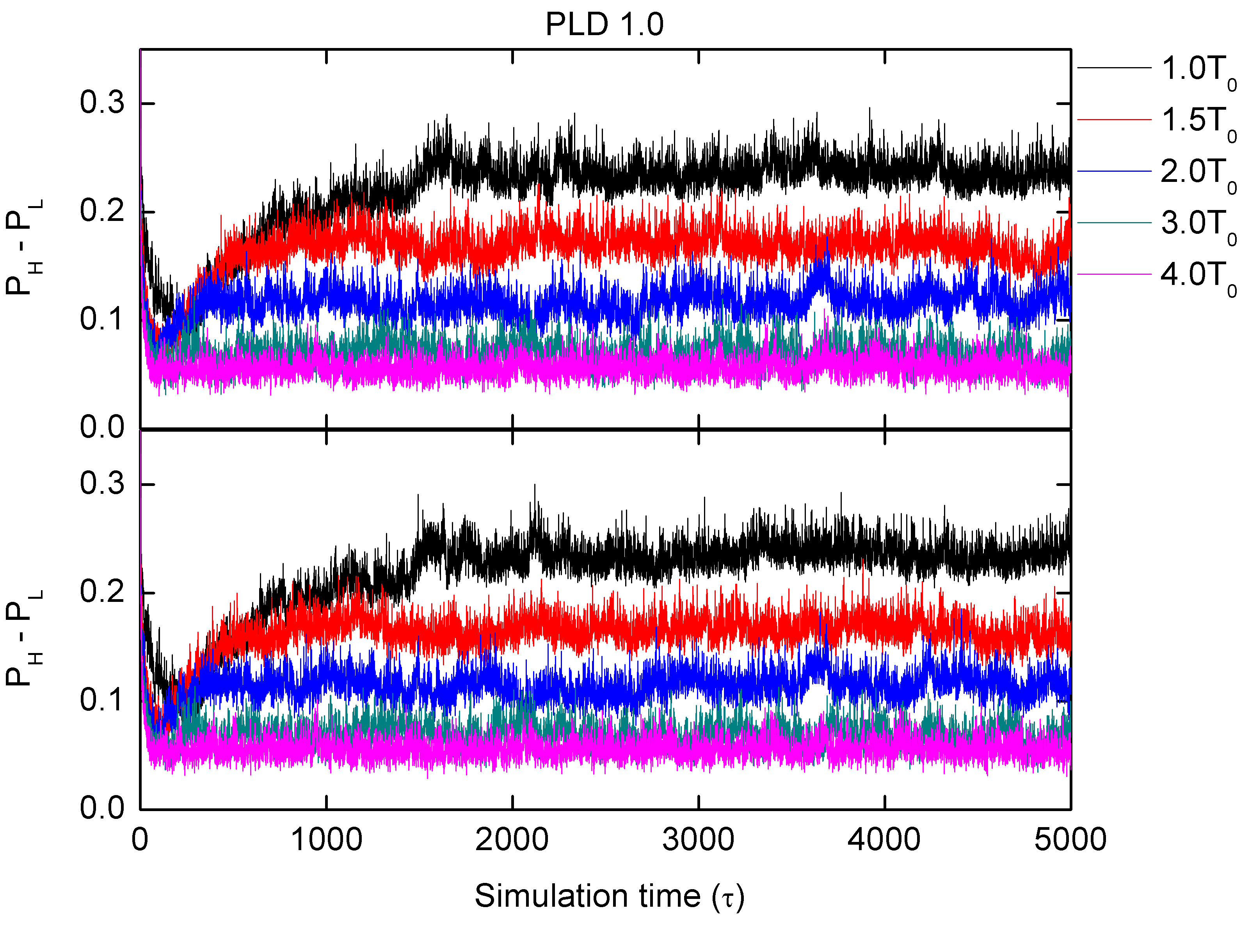

Supplement: Supplementary file 1 [file biomolecules-13-00625-s001.zip › FigS8.PLD1.0.png]

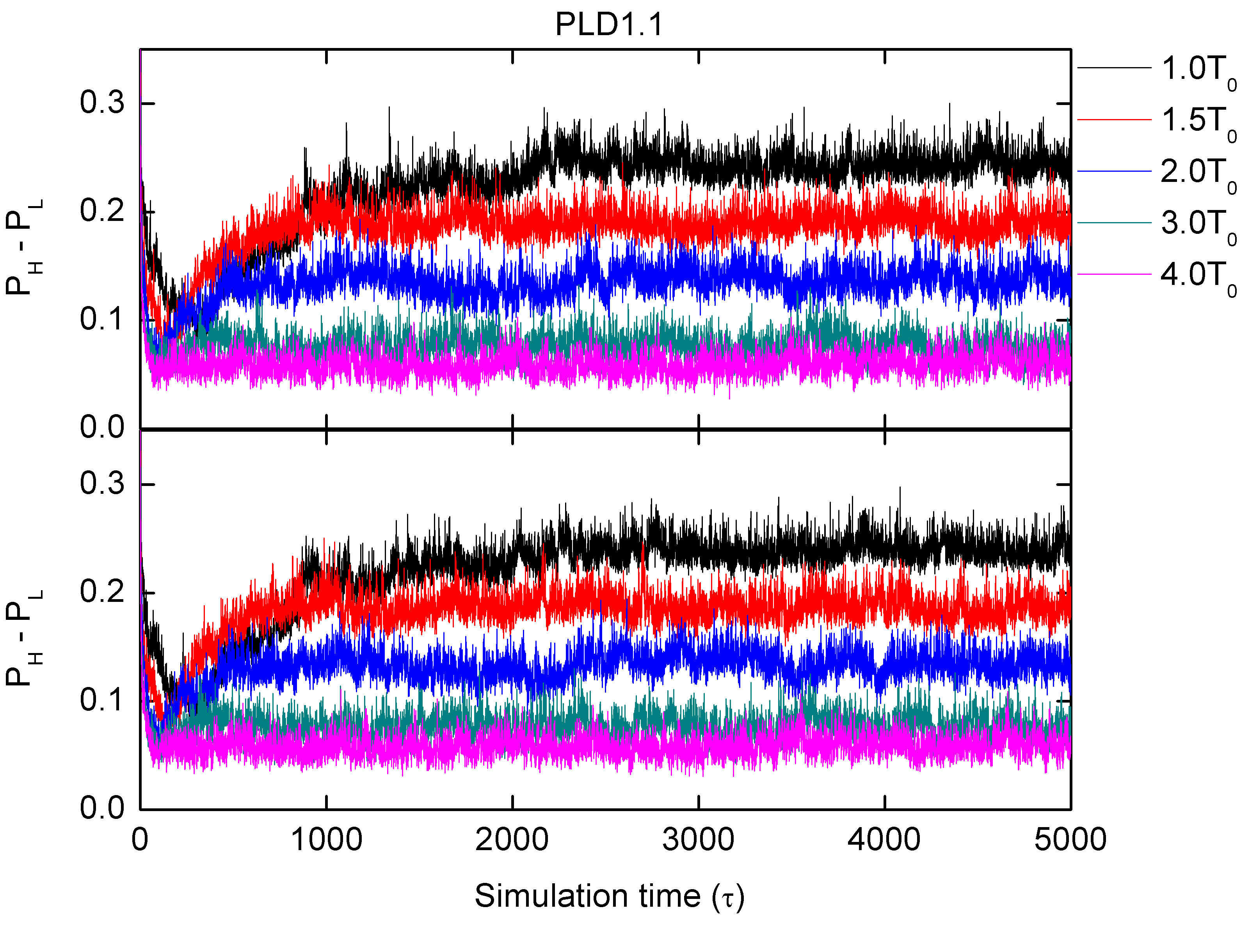

Supplement: Supplementary file 1 [file biomolecules-13-00625-s001.zip › FigS9.PLD1.1.png]
